# Infection-induced extracellular vesicles evoke neuronal transcriptional and epigenetic changes

**DOI:** 10.1038/s41598-023-34074-2

**Published:** 2023-04-27

**Authors:** Ellen Tedford, Norhidayah Binti Badya, Conor Laing, Nozomi Asaoka, Shuji Kaneko, Beatrice Maria Filippi, Glenn Alan McConkey

**Affiliations:** 1 School of Biology, Faculty of Biological Sciences, University of Leeds, Leeds, LS2 9JT UK; 2 Department of Clinical Neurosciences, University of Cambridge, Cambridge, UK; 3 Department of Molecular Pharmacology, Graduate School of Pharmaceutical Sciences, Kyoto University, 46-29 Yoshida-Shimoadachi-Cho, Sakyo-ku, Kyoto, 606-8501 Japan; 4 School of Biomedical Sciences, Faculty of Biological Sciences, University of Leeds, Leeds, UK

**Keywords:** Epigenetics in the nervous system, Parasitology, Gene regulation

## Abstract

Infection with the protozoan *Toxoplasma gondii* induces changes in neurotransmission, neuroinflammation, and behavior, yet it remains elusive how these changes come about. In this study we investigated how norepinephrine levels are altered by infection. TINEV (Toxoplasma-induced neuronal extracellular vesicles) isolated from infected noradrenergic cells down-regulated dopamine ß-hydroxylase (*DBH*) gene expression in human and rodent cells. Here we report that intracerebral injection of TINEVs into the brain is sufficient to induce *DBH* down-regulation and distrupt catecholaminergic signalling. Further, TINEV treatment induced hypermethylation upstream of the DBH gene. An antisense lncRNA to *DBH* was found in purified TINEV preparations. Paracrine signalling to induce transcriptional gene silencing and DNA methylation may be a common mode to regulate neurologic function.

## Introduction

The mechanisms by which intracellular pathogens manipulate their host cells to change signalling pathways and the subsequent impact on host neuronal pathways requires delineation. *Toxoplasma gondii*, an intracellular protozoan in the phylum containing the malaria parasite *Plasmodium*, is a ubiquitous parasite that infects a wide range of warm-blooded animals with approximately one third of the global population seropositive^1^. Seroprevalence in individuals ranges from 10 to 90% in different parts of the world with > 40 m people in the USA seropositive (DPDx, Centers for Disease Control, USA). Acute *T. gondii* infection is followed by a long-standing chronic infection of the brain and muscle that may last years, during which parasites are encysted as bradyzoite stages. In the brain, parasites are encysted exclusively in neurons. Inflammatory responses such as interferon gamma suppress active tachyzoite reproduction with major histocompatibility complex (MHC) I-dependent CD8+ T-cell recognition of infected neurons. Suppression of parasite growth in this way maintains the chronic bradyzoite infection in the brain^2–4^. Changes in behaviour as well as neurotransmission and neurologic function have been observed during chronic infection and correlated with changes in dopamine, norepinephrine, glutamate and GABA.^5–13^. In rodents, loss of innate fear and increased activity favour parasite transmission to the definitive feline host^14–16^. Increased risk-taking and deficits in learning and memory have also been associated with *T. gondii* infection in animal models and human subjects^17–19^. *T. gondii* seroprevalence and serointensity has been correlated with neurological disorders including depression, ADHD, epileptic seizures, movement disorders, and prominently with schizophrenia^3,20–23^.

The mechanisms responsible for neurologic and cognitive changes during infection have only been weakly described at the neurophysiological and gene expression levels. Recently, decreased norepinephrine (NE) concentrations and dopamine ß-hydroxylase (DBH) down-regulation, responsible for NE synthesis, have been observed in the brains of infected rodens and correlated with behaviour changes^7,11^. *DBH* expression was down-regulated 32 ± 2.1-fold relative to uninfected rats (*p* = 0.0023)^7,11^. In that study, lower arousal measured as decreased locomotor activity at early time points (*p* < 0.0001) was observed in infected mice and correlated with *DBH* expression. Increased sociability in infected mice, measured as social approach, also correlated with *DBH* expression. Arousal and sociability are noradrenergic-associated behaviours^7,11,24,25^. As NE also suppresses inflammatory cytokines^26,27^, decreases in concentration would augment the neuroinflammatory response^28^. The observable changes in NE are surprising given the low percentage of cells infected. In a large study investigating brain cysts in rats, only 0.002–0.14% of cells contained tissue cysts for eleven *T. gondii* strains tested^29^. In this study, we addressed the biological questions of how *T. gondii* infection decreases brain NE levels when so few neurons contain the parasite and the mechanism responsible for NE down-regulation. Initial experiments indicated a diffusible factor released by infected cells. EVs were isolated that induced transcriptional gene silencing (TGS) and chromatin remodelling.

## Results

### Toxoplasma infection induces release of extracellular vesicles from host cells that decrease NE

**Figure 1:**
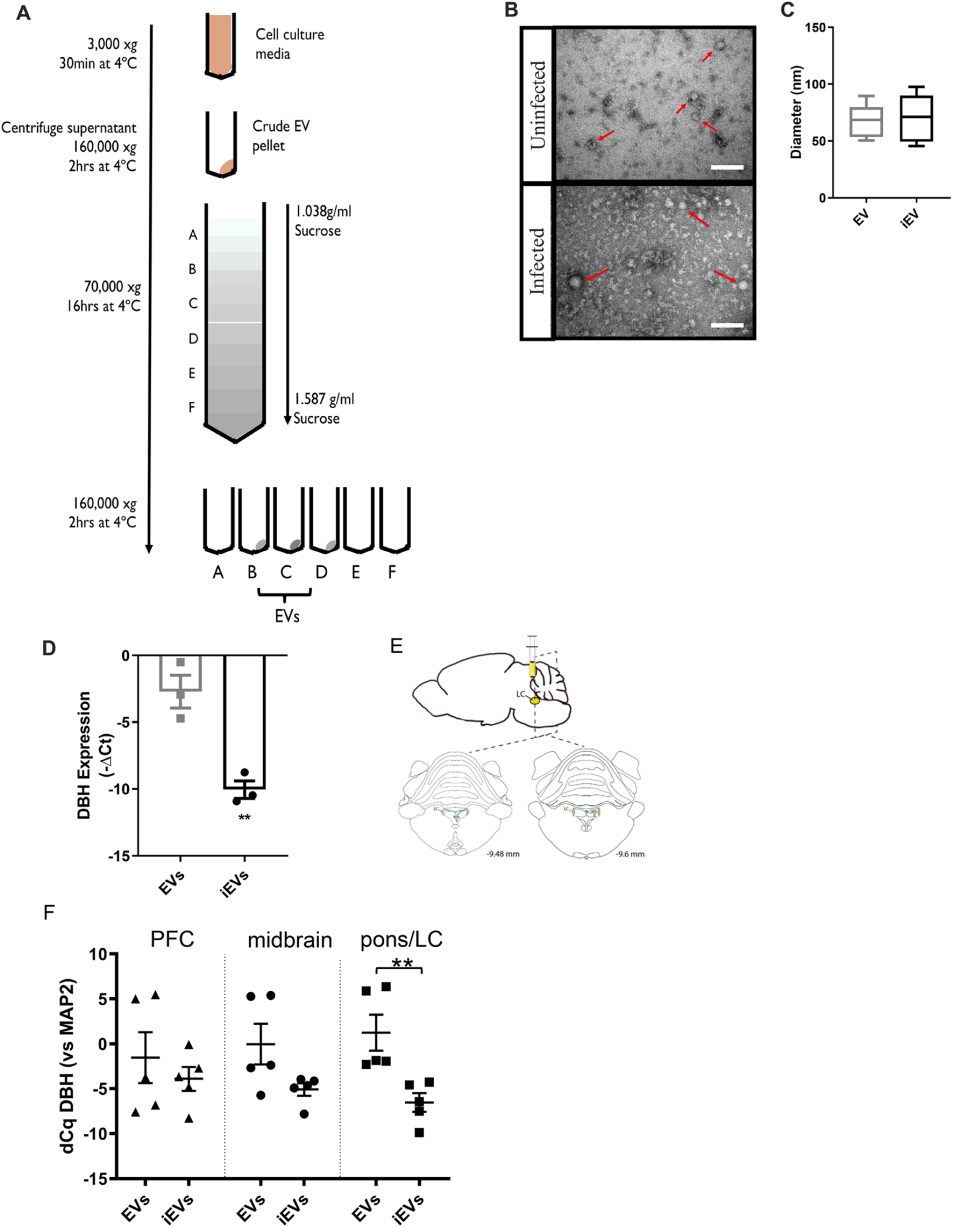
Toxoplasma-induced neuronal extracellular vesicles (TINEV) induce transcriptional regulation in neuronal cells.** (A)** Scheme summarising sequential ultracentrifugation steps undertaken to produce purified extracellular vesicles. Sucrose gradient densities range from 0.104 to 1.43 g/ml. The density between 1.15 and 1.19 g/ml was collected as this represents extracellular vesicles^80^. **(B)** Transmission electron microscopy of extracellular vesicles isolated from uninfected (top) and infected (bottom) PC12 cell cultures. Arrows indicate extracellular vesicles; scale bar represents 100 nm. **(C)** Plot of extracellular vesicle diameter from uninfected (EV) and *T. gondii* infected PC12 cultures (iEV); *p* = 0.81 **(D)** *DBH* mRNA expression following treatment with purified TINEVs. PC12 cells were treated with EVs from uninfected (grey) and *T. gondii*-infected (black) cultures for 24 h; n = 3, **p = 0.006. Graphs show ± SEM with p values for Student’s t tests. **(E)** A schematic representation of experimental design illustrating the site of injection for delivery of EVs directly into the brain’s noradrenergic center, the locus coeruleus (LC), of rats. Brain regions (prefrontal cortex, midbrain, and the pons/LC as illustrated by the dotted lines) were harvested two days following 3 days of twice daily treatment. RNA was purified from tissues and RT-qPCR performed to quantitate *DBH* expression. Stereotaxic coordinates from Paxinos and Watson^81^. **(F)** *DBH* mRNA levels in brain tissues of rats receiving TINEVs (iEVs) and uninfected cell EVs, relative to the neuronal marker MAP2. The pons containing locus coeruleus (pons/LC) contained significantly down-regulated *DBH*. ± SEM shown, n = 5, Mann–Whitney unpaired t test, **p = 0.0079.

Extracellular vesicles (EVs) have emerged as an important facet of host–pathogen interactions and were investigated for their relationship to the previously observed NE suppression^30,31^. EVs were purified from infected catecholamine-producing dopaminergic/noradrenergic cell cultures (henceforth termed noradrenergic cells) by step-wise ultracentrifugation followed by purification by sucrose-gradient fractionation^32,33^ (Fig. 1A). The EVs appear to be of host cell origin based on size, morphology, sedimentation rate, and exosome markers^34^. The infected culture EVs contained mammalian exosomal protein markers CD81 and EpCAM (Fig. S1 and proteomic analysis, Supplementary Table 1) as previously found with PC12 cells, as well as CD63, ICAM, FLOT-1, and TSG101 being detectable. The protein profiles were similar for EVs from infected and uninfected cultures (Fig. S1) and the size was consistent with exosomes (Fig. 1B, C). Based on these properties, in this paper we have termed these *Toxoplasma*-induced neuronal host-derived extracellular vesicles, TINEVs, (also named iEVs). Similar yields of released EVs were isolated regardless of infectious status in our experiments (5.6 ± 1.4 μg/ml and 3.9 ± 0.49 μg/ml, respectively; *p* = 0.438). Uninfected noradrenergic cell cultures were treated with TINEVs and expression of *DBH* was measured. Noradrenergic cells in medium with commercial exosome-depleted serum served as controls. *DBH* expression was significantly down-regulated (190 ± 67-fold, *p* = 0.006 in Fig. 1D) in cultures treated with TINEVs.

Next the in vivo effect of the TINEVs was examined. Intracranial injection of TINEVs into the *locus coeruleus* (LC), the central region of noradrenergic neurons in the brain, of adult rats was performed and *DBH* expression was measured by RT-qPCR (Fig. 1E, Fig. S2). TINEV injection induced a decrease in *DBH* mRNA (62 ± 43-fold, *p* = 0.0079) in the pons/LC region (Fig. 1F) compared to treatment with EVs from uninfected cultures. No difference was found in *DBH* mRNA levels in the mid-brain and prefrontal cortex for rats injected TINEVs, although low numbers of noradrenergic neurons are found in these regions. Health parameters (body weight, appearance and food intake) were normal in treated animals and expression of a housekeeping gene was unaltered in the brain with treatment (Fig. S3). Further, *DBH* expression in the adrenal glands was unaffected by the treatments (data not shown). The decrease in *DBH* mRNA observed with intracranial TINEV injection was similar to that observed in chronic infections^11^.

Levels of NE and *DBH* mRNA have been found decreased with chronic *T. gondii* infection^11^. A direct correlation between *DBH* expression and NE level (Fig. S4; correlation coefficient 0.81, *p* = 0.014) was observed in rodent brains. Hence *DBH* expression can be used as a correlate of brain NE, as found in other studies^35^. Based on the above findings, *T. gondii* infection induces cells to release TINEVs that are able to down-regulate *DBH* expression and cause a widespread decrease in brain NE.

### Strategy for identifying paracrine signalling in *DBH* down-regulation

**Figure 2:**
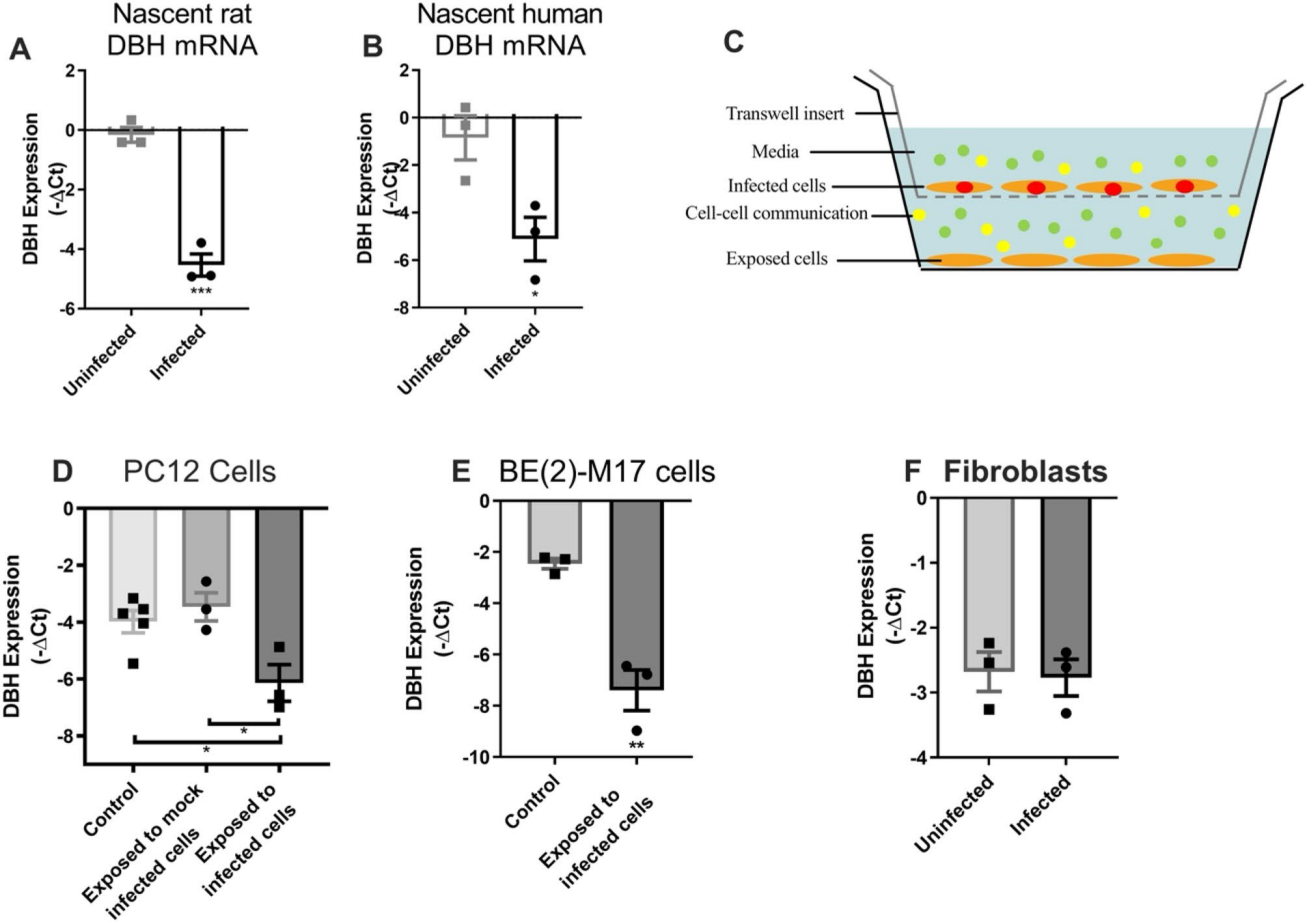
Noradrenergic cells infected with *T. gondii* secrete a signalling factor that transcriptionally regulates *DBH.* **(A)** Nascent *DBH* pre-mRNA transcription (relative to GAPDH) in catecholaminergic PC12 cells infected with pH-shocked parasites. After 5 days of infection nuclear run-on assays were performed. MOI 1; n = 3; ***p < 0.001. **(B)** Nascent *DBH* expression in uninfected and infected human neuronal cells as in (A). Multiplicity of infection (MOI) 1; n = 3, *p < 0.05. **(C)** Scheme of transwell culture system with cells and *T. gondii* on the top layer and uninfected noradrenergic cells on the bottom layer separated by a 0.4 μm membrane. Cells are orange, parasites red, signalling factors in vesicles (green) and soluble (yellow). **(D)** *DBH* expression measured using RT-qPCR (relative to GAPDH) in PC12 cells from the bottom layer with either uninfected control, *T. gondii* infected, or mock-infected cells in the upper transwell layer. Mock-infected cells were incubated with heat-killed *T. gondii* tachyzoites n = 3, *p < 0.05. One-way ANOVA *p* = 0.017, Tukey’s post hoc test uninfected and mock-infected vs infected *p* = 0.020 and *p* = 0.033, respectively. **(E)** Noradrenergic BE(2)-M17 cell expression of *DBH* mRNA cultured in transwells and treated as in (**C**); n = 3, *p* = 0.0038. **(F)** Infected fibroblasts in the upper layer of the transwell system. *DBH* expression levels were measured in exposed noradrenergic cells. *DBH* gene expression was not significantly altered in cells exposed to infected fibroblasts (*p* = 0.84); ± SEM shown, n = 3, Student’s t test.

Initially, it was considered that several different factors could explain the disproportionate decrease in *DBH* expression (and hence NE) relative to the small percent of parasitised cells during chronic infection (e.g. neuroimmune responses). As decreased NE and *DBH* down-regulation (relative to other genes) has been observed in vitro with noradrenergic cell lines, mechanisms other than the host immune system are involved although host cell immunity remained a possibility. A nuclear run-on assay was performed to assess whether the *DBH* down-regulation was at the transcriptional or post-transcriptional level. De novo transcription in nuclei isolated from infected cell cultures was measured by immunocapture of incorporated biotin-UTP. Lower amounts of nascent *DBH* mRNA (relative to standards) were found in host cell nuclei from infected than uninfected noradrenergic cell cultures (21 ± 1.6-fold, *p* = 0.00062) (Fig. 2A). Similarly, de novo transcription of *DBH* was downregulated in infected human noradrenergic cells (21 ± 1.7-fold, *p* = 0.032) (Fig. 2B). Hence, infection induced transcriptional gene silencing (TGS) of *DBH* in human and rat noradrenergic cells.

We then investigated the spreading of *DBH* down-regulation by examining uninfected cells in cultures to determine whether cells exposed to infected cells were also suppressed in NE as this could help explain the large change in expression observed. A transwell system was used that permits uninfected cells to be exposed to *T. gondii*-infected cell products. This method was chosen because it differentiates between diffusible signals and parasites injecting components into cells without invasion, as was observed with a Cre/*loxP* assay in infected mouse brains^36^. *DBH* expression was measured in uninfected rat noradrenergic cells in the bottom reservoir of the transwell system with the top reservoir containing an infected culture (Fig. 2C). *DBH* expression in the cells exposed to infected cultures was found to be down-regulated (7.9 ± 2.8-fold, *p* = 0.02) suggesting that a transmissible factor was released from infected cells. This was subsequently identified as EVs. In contrast, noradrenergic cells that were exposed to cultures containing heat-killed *T. gondii* (‘mock-infected’) were unchanged in *DBH* expression (Fig. 2D). Exposure of a human neuronal cell line to infected cells in the transwell system induced a larger decrease in *DBH* expression (37± 16-fold, *p* = 0.0038) than the rat cell line (Fig. 2E). The observed *DBH* down-regulation is likely to be a minimal baseline as it remains possible that vesicles may stick to the transwell membrane and that EV passage is restricted. Parasite restriction to the upper reservoir of the 0.4 µm filter transwells was confirmed by inoculating standard HFF cultures with media removed from upper and lower reservoirs and monitoring propagation (data not shown). Transwells were set up with infected fibroblasts in the top reservoir and uninfected noradrenergic cells in the bottom reservoir to assess whether the down-regulation was cell-type specific (Fig. 2F). The noradrenergic cells exposed to *T. gondii*-infected fibroblast cultures were unchanged in *DBH* gene expression.

A further indication that EVs were the permeable effector responsible for the *DBH* down-regulation was finding that the insoluble components, separated from soluble factors by ultracentrifugation, contained the *DBH* down-regulating activity in preliminary tests (data not shown). This provided the rationale for EV isolation and testing.

### Epigenetic changes associated with *DBH* down-regulation during infection

**Figure 3:**
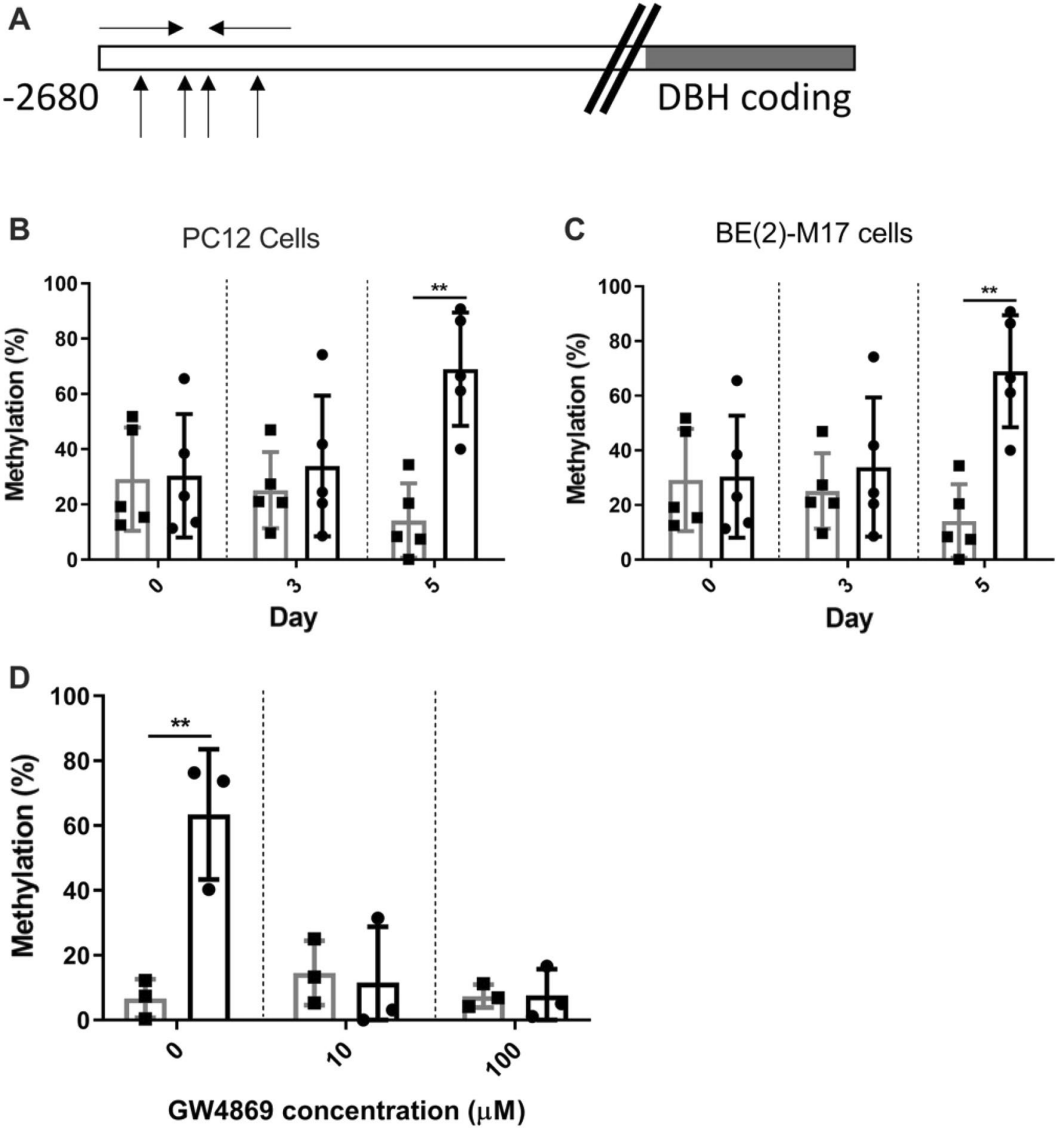
The promoter region of the DBH gene is hypermethylated during *T. gondii* infection.** (A)** Diagram depicting the *DBH* promoter analysis by Methylation Sensitive Restriction Enzyme qPCR (MSRE-qPCR). The DBH coding region is shaded grey. The location of the methylation sensitive restriction enzyme sites are shown with up arrows and the primer sites with facing arrows (not to scale). **(B)** *DBH* promoter methylation was measured as a percentage of methylation in the 5’ region in PC12 cells as measured by MSRE-qPCR. DNA was collected at 0, 3 and 5 days post-infection from uninfected (grey) or infected (black) cultures. MOI = 1; n = 5, ***p = 0.0007. **(C)** Methylation measured as in (**B**) during course of infection for human neuronal (BE(2)-M17) cells. MOI = 1; n = 5, **p = 0.0011. Unpaired Student’s t tests, ± SEM shown. **(D)** Treatment with GW4869, a neutral sphingomyelinase (N-SMase) inhibitor, disrupted the DBH promoter methylation by permeable factors in the transwell system. DBH promoter methylation in the lower layer exposed cells was quantified by MSRE qPCR as described above. ± SEM shown, n = 3, Student’s t test, **p = 0.0093.

As our findings indicated that TGS was responsible for *DBH* expression changes, the epigenetic state of the *DBH* gene was investigated^37^. Methylation Sensitive Restriction Enzyme qPCR (MSRE-qPCR) was used to monitor DNA methylation levels in the *DBH* gene’s upstream region where the majority of CpGs are clustered (Fig. 3A). Methylation in the *DBH* upstream region rose from 16 ± 4.6% to 66 ± 3.8% in infected cultures of noradrenergic cells during the course of the infection (Fig. 3B; *p* = 0.00072). As the noradrenergic cells are sensitive to pH changes (ie. neurotransmitter synthesis and synaptic transmission affected)^38,39^, alkaline-shocked tachyzoites were used for in vitro infections, as in prior studies^40^. This procedure elevated expression of bradyzoite markers BAG1 and SAG4 (Fig. S5). *DBH* methylation was also increased in infected human noradrenergic cultures (2.8-fold; range 2.3–4.8-fold; *p* = 0.0011) with a time-dependent increase in methylation in the region profiled (Fig. 3C).

As an indicator of EV involvement in the epigenetic changes, cells were treated with an inhibitor of EV biogenesis in the transwell system to restrict parasites and parasite-infected cells from contact with the uninfected cell culture layer. The cells exposed to infected cultures had > 50% higher methylation of the *DBH* promoter (Fig. 3D). These cultures were treated with GW4869, a sphingomyelinase inhibitor that disrupts vesicle budding required for endosomal formation. Addition on GW4869 to the transwell system abrogated the DBH hypermethylation observed in uninfected noradrenergic cells exposed to infected cultures.

**Figure 4:**
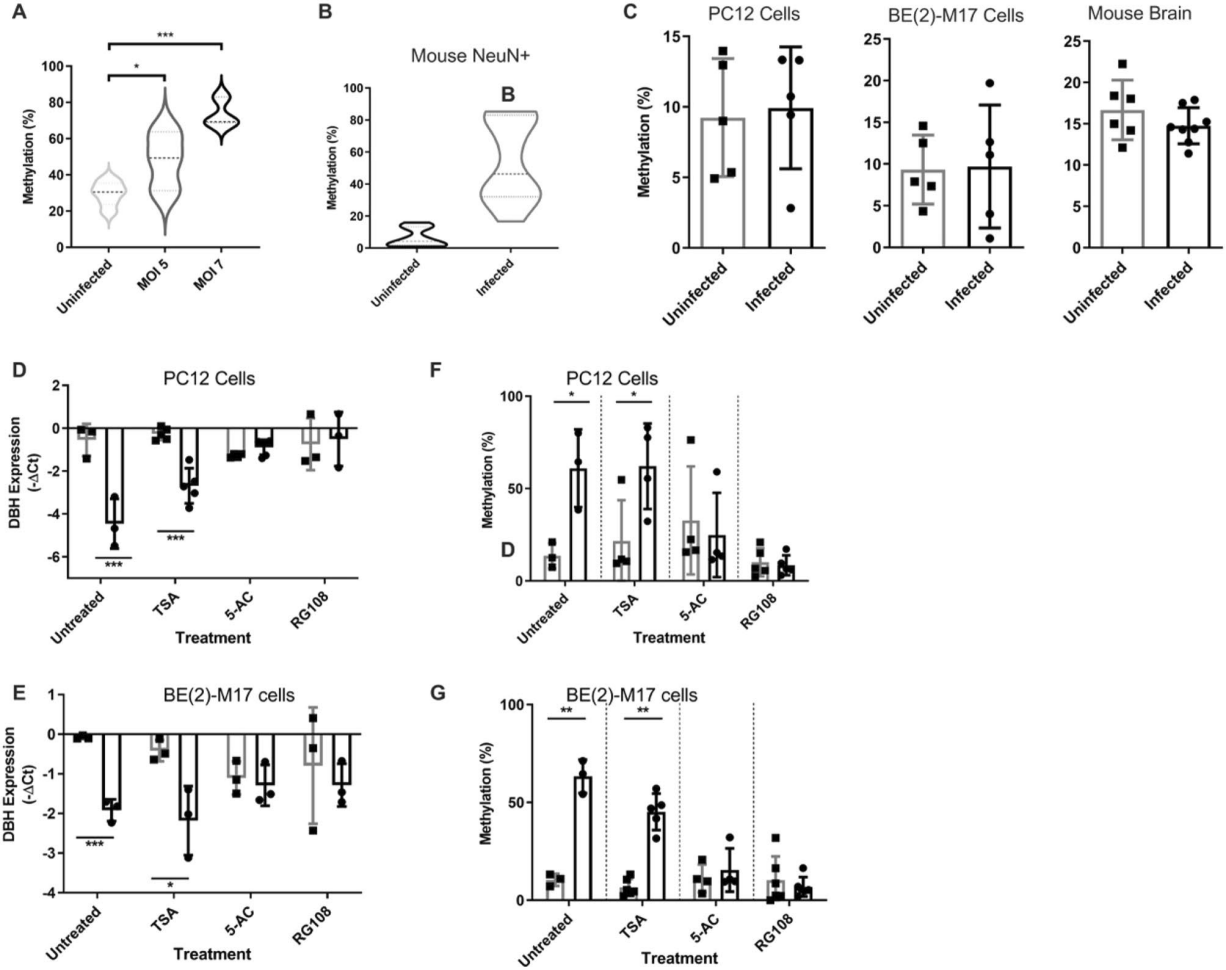
Transcriptional and DNA methylation changes with infection.** (A)** *DBH* promoter methylation was measured by MSRE-qPCR in organotypic coronal midbrain section cultures. Slice cultures were either uninfected (light grey, n = 6) or infected with wild-type induced-bradyzoite *T. gondii* at MOI 5 (dark grey, n = 3) and MOI 7 (black, n = 3). Infection was monitored for 5 days by light microscopy; biological repeats contain 3 slices from the same rat * *p* = 0.04, ***p = 0.000051 shown. One-way ANOVA, *p* = 0.0005 Tukey’s post hoc test MOI 7 vs uninfected *p* = 0.0004. **(B)** Methylation at the *DBH* promoter region in neurons from uninfected (grey, n = 9) and chronically-infected (black, n = 11) mice. Neurons were enriched from harvested brains by FACS using NeuN antibody and *DBH* methylation quantified by MSRE-qPCR. *p* = 0.0004. **(C)** Total genomic DNA methylation measured by ELISA of both uninfected and infected rat catecholaminergic PC12 cells (n = 3, *p* = 0.52), human neuronal BE(2)-M17 cell (n = 5, *p* = 0.59) and neuronal nuclei enriched by FACS from mouse brain tissue for uninfected (n = 6) and chronically-infected mice (n = 3, *p* = 0.15). No significant change in methylation was identified by Student’s t tests, ± SEM shown. **(D)** Expression of *DBH* mRNA measured RT-qPCR relative to GAPDH following treatments. Uninfected (grey) and *T. gondii*-infected (black) noradrenergic PC12 cells treated with trichostatin A (TSA), 5-azacytidine (5-AC) and RG108; n = 5, ** *p* < 0.01. **(E)** As in (**D**) with noradrenergic human BE(2)-M17 cells treated with TSA, 5-AC and RG108; n = 5, *p < 0.05. n = 5. Student’s t test, ± SEM shown. **(F)** 5′ DBH hypermethylation was measured by MSRE-qPCR (as above). Uninfected (grey) and *T. gondii*-infected (black) PC12 cells were treated with trichostatin A (TSA), 5-azacytidine (5-AC) and RG108; n = 5, **p < 0.01. **(G)** Uninfected (grey) and infected (black) BE(2)-M17 cells treated as in (**F**) with TSA, 5-AC and RG108; n = 5, *p < 0.05. Student’s t test, ± SEM shown.

In order to examine the epigenetic effects of *T. gondii* on *DBH* in neurons, ex vivo experiments were performed with infection of organotypic brain slices of the prefrontal cortex, nucleus accumbens and ventral tegmental areas and the TGS in the neuron population measured, as the percentage of infected neurons during chronic infection is 0.002–0.14%^29,41^. Methylation of the *DBH* upstream region rose from 29 ± 2.7% in the brain tissue slices to 74 ± 4.6% in the infected slices (*p* = 0.000051) (Fig. 4A). DNA methylation levels were then analysed in the brains of chronically-infected mice. Neurons were purified from other brain cell types by FACS and *DBH* methylation was measured. The *DBH* gene in infected animals was 53 ± 7.7% methylated in neurons compared to 6.3 ± 2.0% in uninfected mice (*p* = 0.000045, Fig. 4B). For comparison, levels of total genomic DNA methylation were measured (Fig. 4C). No change in global DNA methylation was observed in noradrenergic cells or neurone with *T. gondii* infection, as has previously been found^42^. Hypermethylation of CpG residues upstream of the *DBH* gene were also found by NGS genomic bisulfite sequencing of infected cultures (Fig. S6).

The mechanism responsible for the parasite-induced DNA methylation and chromatin remodelling associated with the silencing was explored by investigating the role of DNA methyltransferase (DNMT) and histone deacetylation in this process. Infected noradrenergic cells were treated with the DNMT inhibitors RG108 and 5-azacytidine (5-AC). Both compounds disrupted the parasite-induced *DBH* down-regulation and DNA hypermethylation in rat noradrenergic cells, relative to marker (Fig. 4D, F). The DNMT inhibitors similarly blocked the TGS and epigenetic changes in human neuronal cells (Fig. 4E, G). This was not due to parasite sensitivity to the inhibitors since the inhibitors were non-toxic to parasites at concentrations tested (data not shown) and *T. gondii* lacks 5-methylcytosine^43^. This implies that the *DBH* silencing involves DNMT. In contrast, the histone deacetylase inhibitor trichostatin A did not abrogate the *DBH* down-regulation or hypermethylation in infected cultures (Fig. 4D–G). Hence the mechanism may not involve histone deacetylation or activation was not captured within the experimental timeframe of 5 days and histone deacetylase is active at a different time in the epigenetic modification pathway.

**Figure 5:**
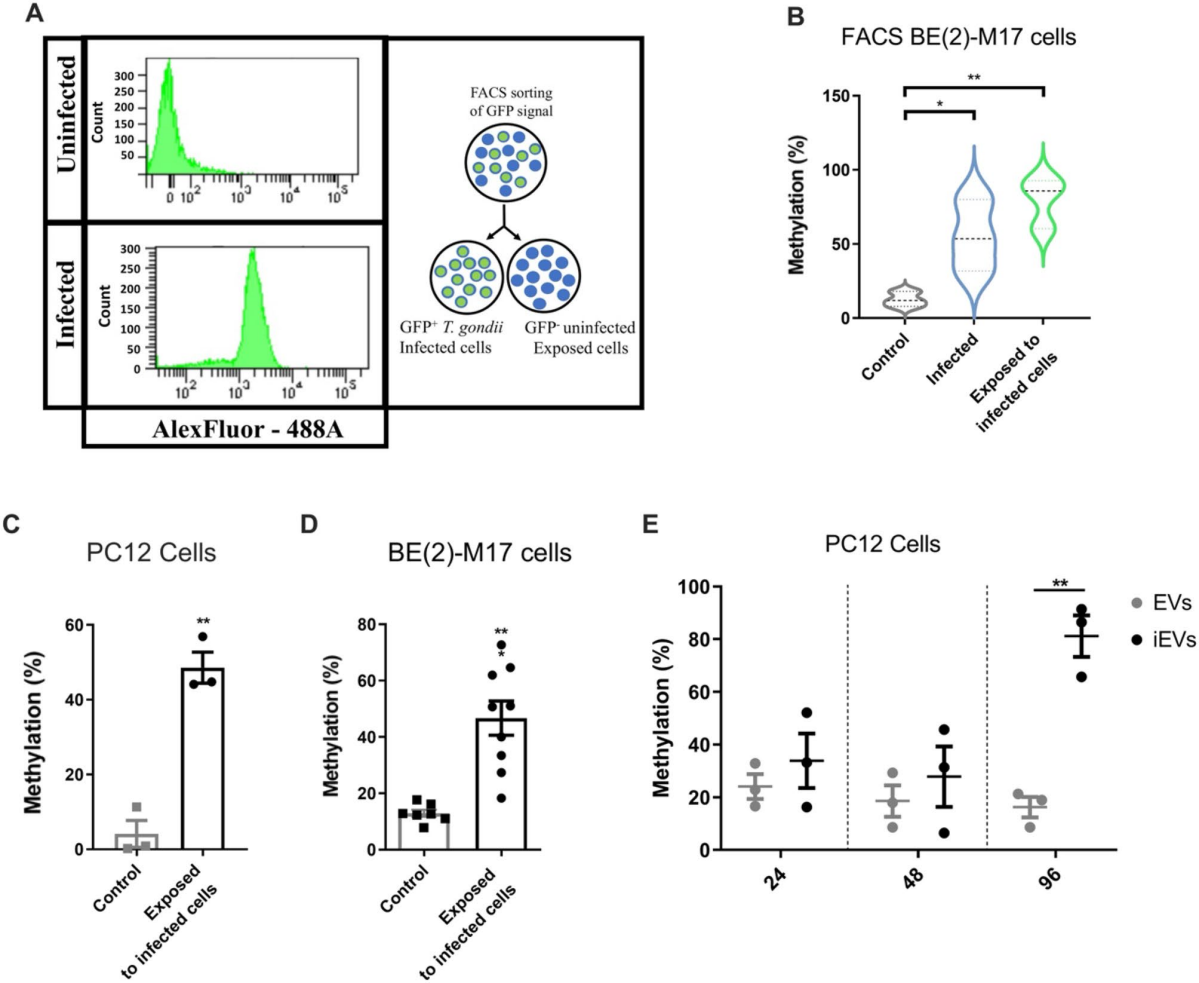
Hypermethylation of the *DBH* gene promoter region in uninfected, exposed cells.** (A)** Representative plot showing cell count against GFP signal for FACs of GFP-negative and GFP-positive cells infected with GFP-expressing parasites. Right panel shows a schematic representation of experimental design. **(B)** Methylation at the 5’ *DBH* region in uninfected cell culture control and GFP+, and GFP(-) FACS-enriched BE(2)-M17 cells measured using MSRE qPCR; n = 3, *p < 0.05, **p < 0.01 shown. One-way ANOVA, *p* = 0.0089, Tukey’s post hoc test exposed to infected cells vs control *p* = 0.0077. **(C)** 5′ *DBH* region methylation in PC12 cells exposed to infected PC12 cells in a transwell system as Fig. 2C. ± SEM shown, n = 3, Student’s t test, **p < 0.01. **(D)** BE(2)-M17 cell *DBH* promoter methylation measured as in (**C**) following 5 days transwell exposure to either uninfected (n = 4) or infected BE(2)-M17 cells (n = 9). ± SEM shown, Student’s t test, ***p < 0.001. **(E)** Time course of methylation changes induced by TINEV treatment. 5’ *DBH* methylation in cells treated with TINEVs (black) and EVs from uninfected cells (grey). Timepoints taken at 24, 48 and 96 h of treatment; Line indicates mean with 95% CI; n = 3, **p = 0.0018.

To directly compare the epigenetic changes in infected versus uninfected (but infection-exposed) cells in the same culture, populations were enriched for cells containing GFP-expressing *T. gondii* (GFP+) and GFP− cells. *DBH* hypermethylation was found in both populations of cells (Fig. 5A, B; ANOVA test, *p* = 0.0089). Indeed, the GFP(-) cells exhibited DNA methylation levels at least equal to the GFP+ cells. This further supports the supposition that the silencing is spread and direct infection is not required for hypermethylation of the *DBH* gene in a cell.

In order to observe the induction of *DBH* hypermethylation in uninfected cells exposed to infected cultures, the methylation status of the *DBH* upstream region in noradrenergic cells was measured by MSRE-sqPCR. Methylation of the *DBH* 5’ region was 34 ± 4.8% greater (*p* = 0.0013) in rat cells exposed to infected cells compared to exposure to uninfected cultures (Fig. 5C). Human noradrenergic cells exposed to infected cells also had increased methylation (49 ± 1.9%, *p* = 0.0003) of the DBH upstream region (Fig. 5D). Hence, both transcriptional down-regulation and promoter hypermethylation are induced in human and rat noradrenergic cells exposed to *T. gondii* cell cultures.

Based on the above findings, the isolated TINEVs ability to induce epigenetic changes was next investigated. *DBH* gene promoter methylation, examined using the MSRE-qPCR assay, increased from 16 ± 3.9% to 81 ± 7.9% over the course of the experiment. In a time course of exposure to TINEVs, methylation was not significantly changed after 24 or 48 h of exposure but hypermethylation was observed at 96 h of incubation (Fig. 5E; ANOVA *p* = 0.0008). Hence *DBH* transcriptional down-regulation and chromatin remodelling are induced by EVs from infected cells.

### Toxoplasma-induced neuronal EVs contain an antisense *DBH* lncRNA

We investigated the RNA cargo for association with the TINEV-induced TGS and epigenetic modifications. Preliminarily, the sensitivity of the TINEVs to ultraviolet (UV) irradiation was tested to assess whether an RNA component could be involved^44^. TINEVs treated with UV radiation no longer induced hypermethylation of the *DBH* promoter (Fig. S7). Although, it is possible that the UV radiation inactivated proteins.

**Figure 6:**
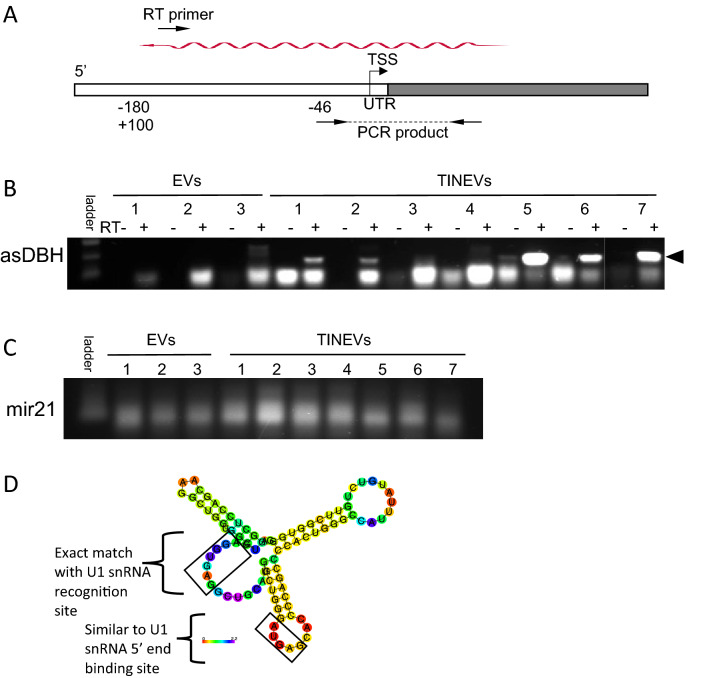
Toxoplasma-induced neuronal extracellular vesicles (TINEV) contain an antisense *DBH* lncRNA. **(A)** Schematic representation of the *DBH* gene. The transcription start site (TSS) and untranslated region are highlighted with the binding sites of the directional RT and PCR primers used to detect the antisense lncRNA. The antisense lncRNA is depicted as the undulating arrow. **(B)** Detection of asDBH by directional RT-PCR. TINEVs purified from repeated experiments (n = 7) and EVs from uninfected cultures (n = 3) resolved by gel electrophoresis with 1 kb Plus DNA Ladder (NEB). The product (arrowhead) is indicated (verified by sequencing). Alternating lanes are control reactions lacking reverse transcriptase. The full gel photo is shown in Fig. S10. **(C)** An agarose gel of RT-PCR of miR-21 from all samples as a control for RNA quality. **(D)** A drawing of the minimum free energy secondary structure prediction of asDBH using RNAfold with the base pair probabilities colored^82^and conserved binding sites for U1 snRNA identified as described in Yin et al.^54^.

With the specificity of the differential gene expression and the role of non-coding RNAs (ncRNA) in regulating gene expression, we explored the potential presence of a long non-coding RNA (lncRNA). LncRNAs have roles in neuronal gene expression such as several highly specific antisense transcripts^45–48^. EVs have been found to contain miRNAs and lncRNAs, although their functional significance is still unclear. With the specificity of the *DBH* down-regulation in *T. gondii* infection and its magnitude with TINEV treatment, the identification of a long non-coding RNA (lncRNA) was investigated^45,49^. A panel of primers walking upstream stepwise from the *DBH* coding region were used to screen for an antisense RNA^37^. These identified a lncRNA in infected cultures (Fig. S8). RNA purified from TINEV preparations were found to contain the *DBH* antisense lncRNA (Fig. 6A–C, Fig. S9, S10). Although the lncRNA location internally as EV cargo is unconfirmed, it is unclear of the functional significance of the location^50^. The lncRNA is complementary to the *DBH* upstream region containing cis-regulatory elements and crosses the transcription start site. As a positive control for samples, miR-21 miRNA served to confirm RNA quality purified from the TINEVs^51^*.* Intriguingly, the timeframe observed for the *DBH* hypermethylation in this study (Fig. 5E) is similar to dynamic DNA methylation changes observed in cells treated with promoter targeted antisense RNA^52^. The observations above represent the first study to show a specific and functionally-relevant lncRNA to be transmitted from one neuronal cell to another regulating neurotransmission^53^.

The *DBH* antisense lncRNA was found to contain the conserved U1 snRNP recognition site that is a consensus sequence identified in chromatin-bound lncRNAs^54^. Further, predicted secondary structure analysis places the U1 snRNP recognition site in a loop region in minimum free energy secondary structures of *DBH* antisense lncRNA (Fig. 6D). In the published study, the recognition site needs to be in a loop region to allow binding of U1 snRNP to the lncRNA to promote chromatin interaction. Future experiments will further investigate this novel induced lncRNA.

## Discussion

Intracellular infection can manipulate gene expression not only within the host cell but signalling to other cells. In this study it was found that *T. gondii* infection resulted in the release of EVs from noradrenergic cells that specifically induced gene silencing and DNA hypermethylation in vitro and in the brain. Many cells of the CNS release EVs including astrocytes, glia, oligodendrocytes, Schwann cells and neurons^55–57^. EV secretion is emerging as an important mechanism of cellular communication within the CNS^58^. TINEVs purified from infected cultures and delivered into the brain induced *DBH* down-regulation in neurons. EVs are continuously released in the brain limiting our ability to detect and isolate those induced by the 0.002–0.14% of cells that are infected during chronic *T. gondii* infection and restricted by the current lack of biomarkers for TINEVs. The findings presented here provide a mechanism that can resolve the enigma of an observable decrease in NE when only a small percentage of brain cells are infected. We propose a model of *T. gondii* inducing TINEV release with host lncRNAs that modulate the host environment^59^.

Studies of extracellular vesicles secreted from pathogen-infected cells, including the apicomplexan parasites, have reported altering the host immune response^60–64^. For example, cells infected with Epstein-Barr virus release EVs containing viral miRNAs that can downregulate cytokine expression in uninfected monocyte-derived dendritic cells. Human miRNAs complexed with Argonaute 2 are released in EVs from Plasmodium-infected red blood cells and induce pro-inflammatory cytokines in macrophages and endothelial cells. The findings reported here, in contrast, demonstrate a role for TINEVs in altering neurophysiology. TINEVs spreading to uninfected neurons carried host mammalian lncRNA, regulated gene expression, and modified chromatin. Intriguingly, NE modulates CNS cytokine release and hence the resultant NE decrease from *DBH* down-regulation will also affect the neuroimmune response. Host hypomethylation of the mammalian arginine vasopressin promoter in the amygdala of *T. gondii*-infected rodents has also been observed^42^.

EV-miRNAs have been reported that alter DNA methylation in surrounding cells^63^. Here, we identified a natural mammalian antisense lncRNA in TINEV preparations. The lncRNA is complimentary to the *DBH* 5’UTR and contains a consensus sequence for chromatin localization. It remains possible that *DBH* down-regulation is imposed by cell-autonomous immunity in noradrenergic cells by another component of TINEVs that has yet to be defined. Chromatin localisation of the lncRNA may occur via U1 snRNP binding at the transcriptionally active *DBH* gene and interacting with RNA polymerase II to disrupt transcription and facilitate DNA methylation. In this study, diffusible products of infected fibroblasts including EVs did not induce *DBH* down-regulation (Fig. 4F). In the future it will be interesting to assess the intercellular crosstalk from infected neurons to different brain cell types and to examine how *T. gondii* induces host cells to express and package selective lncRNAs.

Evidence is rapidly accumulating to support the role of EVs in the regulation of neurogenesis, neuronal connectivity and neuroimmune communication^44,65^. The findings in this study describe a novel form of neurotransmission regulation that may contribute to maintaining chronic infection. We identified a natural antisense lncRNA in TINEV preparations that specifically altered neurotransmitter signalling. In a recent study, reduced CNS noradrenergic signalling during chronic *T. gondii* infection correlated with noradrenergic-linked behaviour changes^11,66^. Chronic infection with bradyzoite-infected neurons permits continuous delivery of TINEVs into the brain. In this study, TINEVs delivered by intracranial injection twice daily for 3-days down-regulated *DBH*. As behaviour changes are measured in rodents during the 4th to 6th week of infection, a sustained supply of TINEVs (or the antisense lncRNA) is necessary for comparative behaviour tests. Also, reduced DBH has been found to elevate dopamine release from LC neurons (such as those modulating learning and memory)^7,67^ which could explain increased dopamine signalling that has been observed with *T. gondii* infection^68^. The neuroimmune system may also be affected by EVs. Changes in NE levels can alter cytokine responses via adrenergic receptors on astrocytes and microglia. Indeed, treatment with an adrenergic receptor agonist reversed the increased locomotion of infected mice^3,69^. Finding that EVs can induce TGS and modify DNA methylation neurons may represent a mechanism, beyond parasitic machinery, in innate neurophysiological function and enhance our understanding of the role of transcriptional regulation and dynamic DNA methylation in memory and learning.

## Methods

### Cell culture

#### Parasite and cell culturing

*T. gondii* Prugniaud strain, previously isolated from mouse neuronal cysts, was used unless otherwise stated. Parasites were maintained in monolayer cultures of human foreskin fibroblasts (HFFs, ATCC® SCRC-1041) in Dulbecco’s Modified Eagles Medium (DMEM, GIBCO, USA) containing 10% foetal bovine serum (FBS) and 20 μg/ml^−1^ penicillin/streptmycin (Sigma, USA). Incubation of free tachyzoites in pH 8.2 DMEM with 5% FBS, overnight at 37 °C was used to induce tachyzoites conversion to bradyzoites as previously described^11^. Expression of *T. gondii* differentiation markers over 5 days of infection confirmed bradyzoite conversion (Figure S5). All experiments were performed with induced parasites unless otherwise stated.

Rat adrenal phaeochromocytoma, PC12, (ATCC® CRL-1721) cells were maintained at a density of 2–8 × 10^5^ cells/ml in Roswell Park Memorial Institute (RPMI) 1640 Medium (GIBCO, USA) and supplemented with 10% horse serum (GIBCO, USA), 5% FBS (GIBCO, USA) and 20 μg/ml^−1^ penicillin streptomycin (Sigma, USA). Human neuronal BE(2)-M17 cells (ATCC® CRL-2267) were maintained in a 1:1 ratio of F12 Hams and OptiMEM (GIBCO, USA) media supplemented with 10% horse serum (GIBCO, USA), 5% FBS (GIBCO, USA) and 20 μg/ml^−1^ penicillin streptomycin (Sigma, USA). The production of dopamine (DA) and norepinephrine (NE) by these cell lines was monitored regularly by HPLC-electrochemical detection. For experiments in which Extracellular vesicles (EV) were harvested or analysed, exosome depleted-FBS (GIBCO, USA) replaced the serum in the media for 24 h prior to harvest. All cells were maintained at 5% carbon dioxide and 37 °C and monitored for growth by light microscopy.

### Infection of PC12 and BE(2)-M17 cells

Cells were cultured in multi-well plates at a density of 5 × 10^4^ cells/ml. When stated, transwell plates (Nunc™ cell culture inserts, Thermo Scientific) were used, with cells plated at 2.5 × 10^4^ cells/ml. Following 24 h of growth on 6-well plates, cells were infected with induced Prugniaud parasites in upper-wells, maintaining a multiplicity of infection (MOI) of 1. Cells were harvested immediately following infection (day 0) and after 3 and 5 days of infection for downstream processing. Mock-infection was performed using heat-killed *T. gondii* parasites subjected to 80 °C for 10 min. The cultures were monitored daily by light microscopy.

### Treatment of cells with purified TINEVs

EVs were extracted and purified from cell medium of uninfected and infected cells in exosome-depleted media, as described below. PC12 cells plated on 6-well plates and grown to a density of 2 × 10^4^ cells/ml were treated with EVs at a 10:1 culture media concentration equivalent (ie. 1 ml of PC12 cell culture treated with EVs purified from 10 ml of culture). Cells were treated every 24 h. DNA and RNA were harvested 24 h after the last treatment.

### Drug assays

Cells were cultured and infected as previously described. Drug was added one hour before the time of infection (day 0). Cultures were grown in media containing a range of inhibitor concentrations; GW4869 (0–100 μM), Trichostatin A (TSA, 0–100 nM), RG108 (0–100 nM), or 5-Azacytidine (5-AC, 0–50 μM) (SIGMA, USA). The effect of drug treatment on the parasites was monitored by infecting human fibroblasts with KU80-GFP bradyzoites or RH-YFP tachyzoites^70,71^ growth was measured by fluorescence using a FLUOstar Omega plate reader (BMG, UK).

### Fluorescence-assisted cell sorting (FACS) of infected human neuronal cells

Human neuronal cells were infected with the Pru ∆hpt/GFP/FLUC *T. gondii* strain, (kind gift from Boothroyd)^72^. Five day infected and uninfected cell cultures were resuspended in PBS and separated in a CytoFLEX–2 flow cytometer (Becton Dickenson, USA) to isolate negative and GFP+ fluorescent cells. Control uninfected cell cultures were also subjected to FACS. DNA was extracted and purified from FACS populations.

### Rodent experiments

Nine-week-old wild-type Sprague Dawley male rats weighing 245 ± 15 g were obtained from Charles River Laboratories (Margate, UK) and were used in line with the United Kingdom Animals (Scientific Procedures) Act 1986 and ethical standards set by the University of Leeds Ethical Review Committee and in accordance with the ARRIVE guidelines. Animals were housed individually and maintained on a 12-h light–dark cycle with access to food and water ad libitum. Rats were monitored for health and fitness parameters of weight and food intake as shown in the supplemental data and the one that did not recover after surgery (lost more than 10% of their body weight) was not used in the experiments. Animals were randomly assigned from a total of 11 rats; 10 were used in this study. Animal care was blinded with investigators aware of the treatments. Animals were treated twice daily for 3 days. The outcome was measured by expression of DBH and compared by Student’s t-test.

Six male Lister Hood rats (Harlan Ltd, UK), 9-weeks old and weighing circa 300 g were infected and monitored as previously described^11^. Rats were housed in individual cages and maintained on a standard light–dark cycle with access to chow diet and water ad libitum. Animals were infected by intraperitoneal (IP) injection with tachyzoites. Throughout the experiments animals were monitored for illness or weight loss (more than 10%) and sacrificed 5–6 months post-infection. Brains were snap-frozen for cryosectioning and RNA extraction.

(BALB/cAnNCrl × C57BL/6NCrl) F1 generation male mice (29 mice, no exclusions) were housed up to five to a cage and maintained on a standard light–dark cycle with access to chow diet and water ad libitum. Throughout the experiments animals were monitored for illness or weight loss (more than 10%) and sacrificed 4–6 weeks after infection in the University of Leeds research animal facility with procedures approved by the University Animal Care and Use Committee and following Home Office, HSE, regulations and guidelines for Animals (Scientific Procedures) Act 1986 published in 2014 and in accordance with ARRIVE guidelines with considerations of the replacement, reduction, and refinement in the use of animals for research. The mouse infections, brain dissection and frozen cryostat sectioning was performed as described^11^.

### Organotypic brain slice cultures

Sprague–Dawley rat pups (one litter) from females housed individually with ad libitum chow diet and water at postnatal days 3–4 were anesthetized as approved, following the Kyoto University Animal Research Committee approved protocols in the Kaneko Laboratory at Kyoto University. Sectioning was performed as described^41^. Brain was removed and separated into two hemispheres. Coronal sections (350 μm thickness) were prepared under sterile conditions. Slices were dissected to include the ventral tegmental area (VTA). These slice cultures were placed on 30 mm insert membranes (Millicell 0.4 μm; Millipore). Slice culture medium contained RPMI supplemented with 10% horse serum (GIBCO, USA), 5% FBS and additional 6.5 mg/ml glucose and 2 mM l-glutamine. The brain slice cultures were cultured at 37 °C in a 5% CO^2^ for 15 days after dissection prior to use in experiments.

### Extracellular vesicle intracerebral treatment

Intracerebral injection into adult rats followed previously described experimental procedures^73^. TINEVs extracted and purified from rat catecholaminergic PC12 cells as described below were used to treat rats. Rats were stereotactically (David Kopf Instruments) implanted with indwelling bilateral cannula targeting the locus coeruleus. After one week of recovery, implanted rats received a 2 μl infusion twice a day for 3 days with TINEVs (4 μg protein) isolated from infected PC12 cells. EVs from uninfected PC12 cells served as a control. On the fourth day rats were anaesthetized and received an injection 3 μl bromophenol blue through each side of the bilateral DVC cannula (to verify their placement) prior to harvesting brain tissue. Brain tissue was dissected into prefrontal cortex, midbrain, and hindbrain regions for processing for RNA.

### Fluorescence-assisted cell sorting (FACS) of mouse neurons

Mouse brain samples were processed as described^74^. Briefly, brains were suspended in lysis buffer (0.32 M sucrose; 0.1 mM ethylenediaminetetraacetic acid; 5 mM CaCl_2_; 3 mM Mg(Acetate)_2_; 10 mM Tris–HCl pH 8; 1 mM dithiothreitol; and 0.1% Triton X-100) and processed using a 15 ml glass dounce homogenizer (Wheaton, UK) in 5–10 strokes on ice. Samples were centrifuged at 100,000xg with a two-step sucrose gradient to purify nuclei. Nuclei were stained with primary antibody NeuN (Abcam, EPR12763 1:250) and secondary antibody Alexa-488 (Abcam, ab150077, 1:1000). All nuclei were also stained with Hoechst 33342 (SIGMA, USA). Samples were sorted using the Becton Dickinson FACSAria II system.

### Extracellular vesicle characterization

#### Extracellular vesicle purification

TINEVs were isolated from media by ultracentrifugation as described^75,76^. For all experiments, EVs were isolated in parallel from uninfected cells. Briefly, one day prior to harvest, complete media was replaced with media containing exosome-depleted FBS (Systems Bioscience, CA). Cell cultures of uninfected and infected cells (five days following infection) were harvested and centrifuged at 3000×*g* for 10 min at 4 °C. The supernatant was isolated and centrifuged at 160,000×*g* for 2 h in a Type 60 Ti fixed angle rotor at 4 °C. The pellet was resuspended in one ml of 90% sucrose. Eleven layers of one ml of sucrose 70%-10% w/v were then added and centrifuged at 70,000×*g* for 16 h at 4 °C in a Type 60 Ti fixed angle rotor. Supernatant was collected in two ml fractions. PBS (8 ml) was added to the fraction 1.1–1.2 g/ml sucrose with a density corresponding to that of EVs and centrifuged at 160,000xg for 70 min at 4 °C to recover purified EVs. Methods and data on purification and characterization were input to the EV-TRACK knowledgebase (EV-TRACK ID: EV220363) (https://evtrack.org/).

### UV treatment

EVs were isolated as described. Prior to UV treatment, *DBH* down-regulation by TINEVs was verified following incubation of noradrenergic cells for 24 h. TINEVs were exposed or mock exposed to UV light at 302 nm for 5 min at 4 °C using a transilluminator (Syngene, Cambridge).

### Dot blotting

Dot blotting was performed using the Exo-Check Exosome Antibody Array (Systems Bioscience, CA) as per manufacturer's instructions. Briefly, 500 μg protein equivalent of purified EVs isolated from PC12 culture were lysed and ligated to horseradish peroxidase (HRP) overnight at 4 °C. Ligated protein was then incubated with the antibody membrane for two hours at room temperature. After three washes SuperSignal West Femto Chemiluminescent Substrate kit (Thermo Scientific, UK) was used as per manufacturer's instructions. The blot was visualised via 90 s exposure to X ray film. Film was developed using the Konica SRX-101A Tabletop Processor.

### Transmission electron microscopy (TEM)

EVs were purified as described above. Electron microscopy was performed as described^77^. Freshly isolated EVs were resuspended in cold Dulbecco Phosphate-Buffered Saline (DPBS) containing 2% paraformaldehyde. EVs were mounted on copper grids, fixed with 1% glutaraldehyde in cold DPBS for 5 min at room temperature, negative stain was performed with uranyl-oxalate solution at pH 7 for 5 min, and embedded with methyl cellulose-UA for 10 min at 4 °C. Excess cellulose was removed, and samples were dried for permanent preservation. Electron microscopy was performed in the University of Leeds facility using a Titan Krios 2 electron microscope. Analysis and extracellular vesicle diameter were measured using ImageJ software.

### Molecular techniques

#### DNA extraction

Classic phenol–chloroform method extraction was performed as described in^78^. Briefly, tissue samples were first homogenised using a Micro Tissue Homogenizer (Fisher Scientific, UK). Homogenised tissue or cell pellets were incubated at 56 °C overnight with 20 mg/ml Proteinase K. An equal amount of phenol/chloroform/isoamyl alcohol (PCI) solution (25:24:1) was then added, mixed and centrifuged. The aqueous layer was isolated and an equal volume of chloroform:isoamyl (CI) alcohol (24:1) added. DNA was precipitated using absolute ethanol and DNA, then resuspended in deionised water.

RNA extraction was performed using Direct-zol (Zymo, USA) as per manufacturer’s instructions.

### PCR

Polymerase chain reaction was performed using the GoTaq PCR master mix (Promega, USA) as per manufacturer’s instructions. Samples were amplified in a 2720 thermal cycler (Applied Biosystems, USA). The PCR parameters were 94 °C for 30 s; 35 cycles of 94 °C for 30 s (denaturing step), 55 °C for 15 s (annealing step) and 72 °C for 10 s (elongation step); a final elongation of 72 °C for 30 s.

Samples were visualised on a 2% w/v agarose gel with a Trackit 1 kb Plus DNA ladder run at 80 V until the DNA ladder and the sample products of interest had separated adequately.

### RT-qPCR

RT-qPCR was performed using SYBR green master mix (Life Technologies, USA) as per manufacturer’s instructions and run using a CFX Max Real Time PCR machine (Bio-rad, USA). Parameters were 95 °C for 2 min and followed by 45 cycles of 95 °C for 15 s, (primer Tm-5) °C for 15 s, and 72 °C for 10 s. Primer sequences are recorded in Supplementary Table 2. A melt-curve was performed to check for expected products. All RT-qPCR was performed with four technical replicates which were averaged.

### Nuclear run-on

Nuclear run-on was performed as described^79^. Briefly, after five days of infection, cells were incubated with lysis buffer containing 10 mM Tris–HCl pH 7.4, 10 mM NaCl, 3 mM MgCl2, 0.5% NP-40 at 4 °C isolating nuclei. Nuclei are then suspended in transcription buffer containing 20 mM Tris-HCl pH 8.3, 5 mM MgCl2, 300 mM KCl, 4 mM DTT, RNase OUT 40 Units/μl, 0.5 mM BrUTP, 1 mM ATP, 1 mM GTP, 1 mM CTP, 1 mM UTP. Samples were incubated for 45 min at 37 °C prior to RNA extraction using MEGAclear Transcription Clean-Up Kit (Life Technologies) as per manufacturer’s instructions. Bead-based immunoprecipitation was performed with protein G Dynabeads and IgG anti-BrdU antibody IIB5 (Santa Cruz Biotechnology, SC-32323). RNA was extracted and RT-qPCR performed as previously described.

### Methylation sensitive restriction enzyme (MSRE) quantitative PCR

DNA was extracted from cell cultures and isolated as previously described. DNA concentrations were confirmed using a Nanodrop spectrophotometer. Samples were divided into reference and test samples. Test samples were digested with the high-fidelity methylation sensitive restriction enzymes (MSRE) HpaII, MaeII and SmaI (TaiI) in the Tango buffer (Thermo Fisher Scientific, USA). These MSREs were chosen based on the restriction enzymes sites present in the DBH upstream region. Reference samples were mock digested. Digestion was performed by heating to 25 °C for 30 min, 37° for 30 min and 65° for 30 min. RT-qPCR was then performed. Percentage methylation calculated based on the difference in Cq values between test and reference samples. Digestion was confirmed with methylated and unmethylated human DNA standards provided with the OneStep qMethyl kit (Zymo) as per manufacturer’s instructions. Additionally, primers and MSRE digestion was confirmed with MSRE-PCR. qPCR was performed using SYBR®Green Real-Time PCR Master Mix (Thermo Fisher).

### Directional RT-PCR for DBH lncRNA screening

Three single-stranded forward (i.e. sense) directional primers were designed to detect antisense RNA and are labelled RT-Primer 1, RT-Primer 2, and RT-Primer 3 (Extended Data Fig. 6A). The RT-designed primers are located (1) in the first exon, (2) near the transcription start site, and (3) − 378 bp upstream of the DBH coding sequence. Four pairs of PCR primers were then designed to amplify products from the RT primer synthesized-template to screen for the presence of antisense lncRNA (Extended Data Fig. 6A). A list of primers used is shown, Supplementary Table 2.

Total RNA was extracted from *T. gondii* infected-PC12 cells that were harvested on day five post-infection using the Direct-zol RNA MiniPrep Plus kit (Zymo Research, USA) according to the manufacturer’s instructions. DNase I treatment was implemented as suggested by the manufacturer. In addition, to ensure that all trace amounts of DNA contamination were removed, the eluted RNA samples were treated again with DNase enzyme using TURBO DNA-free™ kit (Invitrogen, USA) according to the manufacturer's instructions as in earlier studies^37^. The dependence of PCR products on the RNA samples was verified by parallel experiments with RNase treatment (data not shown). RNA samples were reverse transcribed with first strand synthesis primed with RT-primer 1 to 3 with a Maxima H Minus First Strand cDNA synthesis kit (Thermo Scientific, USA) following the manufacturer’s instructions. For each of the samples, there were several controls including priming the cDNA synthesis with random hexamer primer, a negative control reaction to assess the gDNA contamination in the RNA sample containing all components for RT-PCR except the Maxima H Minus enzyme mix, and no template control to assess reagent contamination. The mixtures were incubated as follows: 25 °C for 10 min, 50 °C for 30 min, 65 °C for 20 min and then the reaction was terminated by heating at 85 °C for 5 min. The reaction products served as templates for PCR with GoTaq ® G2 Hot Start Master Mix (Promega, USA), 300 nM forward primer, 300 nM reverse primer, and 2 μl template DNA. Thermal cycling was 3 min at 95 °C, followed by 30 cycles of 95 °C for 30 s, 57 °C for 30 s, 72 °C for 20 s and final termination at 72 °C for 5 min in a thermocycler (Applied Biosystems, USA). All PCR products were resolved and visualized by 1.5% to 2% w/v agarose gel electrophoresis. For DNA sequencing, the specific band was gel-excised and gel-extracted using QIAQuick Gel Extraction Kit (Qiagen, Germany). The eluted DNA concentrations were then measured by the NanoDrop spectrophotometer (Thermo Fisher Scientific, UK). The PCR products were subcloned into a TOPO TA cloning vector (PCRTM4-TOPO–Invitrogen TA cloning kit, Thermo Fisher Scientific, UK) and transformed into XL-10 Gold ultracompetent cells (Agilent Technologies, USA). Two clones bearing inserts of each sample were sent for Sanger Sequencing to Genewiz, UK.

### Global methylation

Global methylation was measured using a colourimetric, ELISA adapted method, MethylFlash Methylated DNA 5-mC Quantification Kit (Epigentek, US) as per manufacturer's instructions. DNA (5 ng) was used for quantification and absorbance (405 nm) was read using the Molecular Devices SPECTRA MAX PLUS Plate Reader Ver. 3.05 Spectrophotometer.

### Whole-genome bisulphite sequencing

Whole-genome sequencing of uninfected and *T. gondii*-infected PC12 cells was performed at the University of Leeds Next Generation Sequencing facility at St James Hospital. DNA samples were collected by phenol–chloroform extraction as previously described. Five biological replicates were pooled for uninfected and control samples. Libraries were prepared using the TruSeq DNA Methylation Kit (Illumina, UK). Briefly, samples were bisulphite treated using the EZ DNA Methylation-Gold Kit (Zymo). Treated DNA was then amplified and 5’ adapters ligated. 3’ indexing adapters were then ligated to the ssDNA and 12 rounds of PCR performed. Sequencing was performed using the HiSeq 4000 Systems (Illumina). Differential methylation between infected and uninfected samples was calculated based on genome alignments by the Facility.

### Statistical analysis

All statistical analysis was performed using GraphPad and SPSS. For each data set, Levene’s test of equal variance was performed. Unless otherwise stated, a Student’s t test and where appropriate ANOVA with a post hoc test were performed for all equally distributed data.

## Supplementary Information

Supplementary Information.

## Data Availability

All data generated and analysed during this study are included in this published article [and its supplementary information files]. The raw datasets from the current study are available from the corresponding author on request.
