# Supplementary material for: Infection-induced extracellular vesicles evoke neuronal transcriptional and epigenetic changes
Source: Sci Rep. 2023 Apr 27;13:6913. doi: 10.1038/s41598-023-34074-2 (PMC10140046; doi:10.1038/s41598-023-34074-2)
Supplement: Supplementary file 1 — Supplementary Information. [file 41598_2023_34074_MOESM1_ESM.docx]

**Infection-Induced Extracellular Vesicles Evoke Transcriptional and Epigenetic Changes in Neurons**

Tedford et al.

**SUPPLEMENTAL INFORMATION**

Day 5 of infection


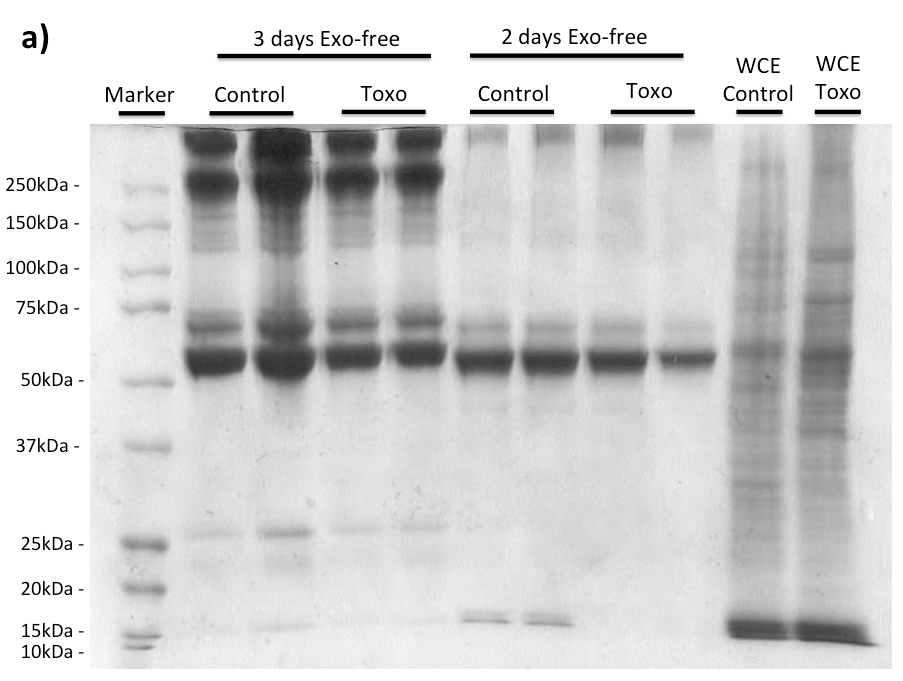


Day 4 of infection

**Figure S1: Extracellular vesicles isolated from infected cells exhibit classical exosomal markers.** **A)** Image of a commercial dot blot probed with protein (300μg) from purified extracellular vesicles from *T. gondii* infected rat catecholaminergic cells. The dot blot contained eight common exosome markers and GM130 to indicate cellular contamination. (Exo-Check™ Exosome Antibody Array, System Biosciences, Palo Alto) with upper left and lower right HRP-conjugated antibody as positive controls. **B)** Quantitation of luminescence in the blot of the exosome markers using imageJ software. **C)** Protein analysis of EVs isolated from PC12 cells and infected cells. Purified protein from EVs was resolved by 12% SDS-PAGE and a coomassie blue stained image is shown. Protein markers and control whole cell extracts (WCE) are shown.

**
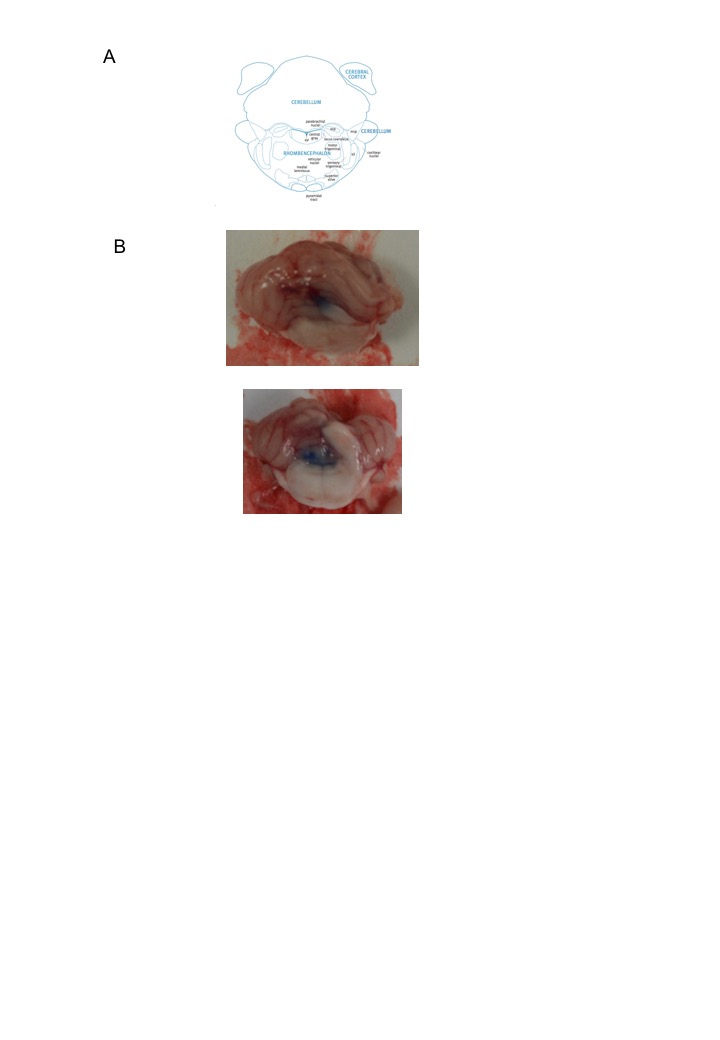
**

**Figure S2: Delivery of EVs to the noradrenergic center, the locus coeruleus. A)** Diagram of coronal section of the rat hindbrain taken from Paxinos and Watson, Academic Press **The Rat Brain in Stereotaxic Coordinates 6^th^ ed. B)** Examples of hindbrains dissected from rats shows localization of EV delivery by the bromophenol blue dye delivered through the cannula.


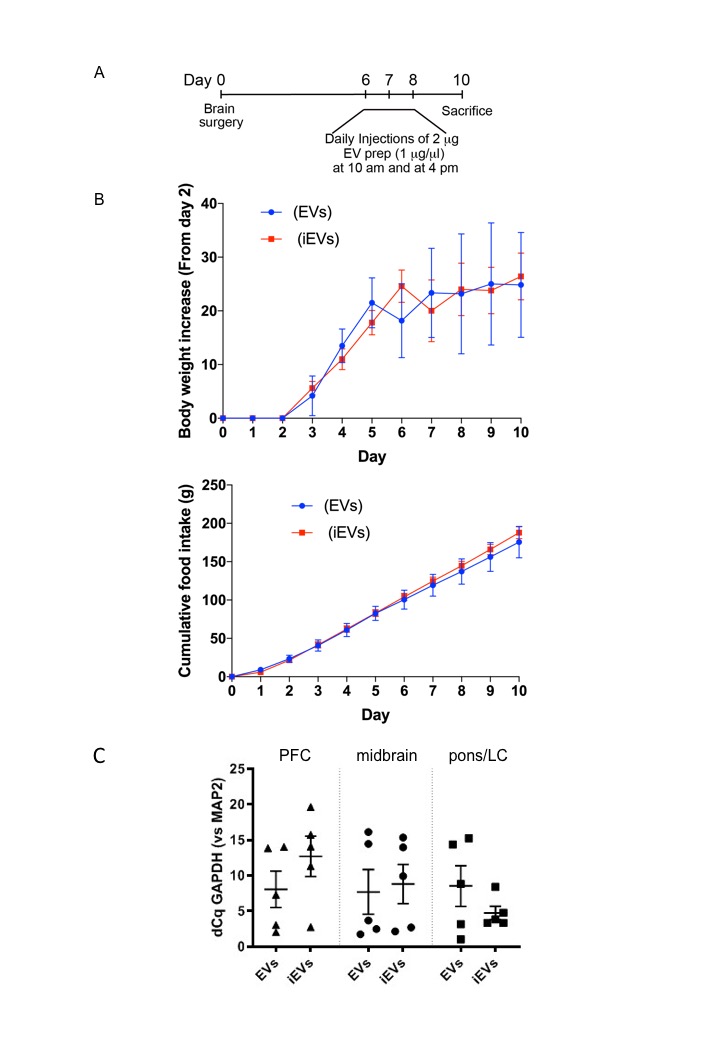


**Figure S3: Intracerebral injection of extracellular vesicles (EVs) effect on health status and brain GAPDH expression. A)** Timeline of the protocol for intracranial treatment of rats with purified EVs **B)** Body weight and appetite, measured as food consumption, in animals; during the course of the experiment. Following recovery from surgery these are normal. **C)** Expression of GAPDH in brain cells following EV treatment. Rats received *T. gondii*-induced neuronal extracellular vesicles (TINEV) labelled as iEV or EVs purified from uninfected cultures. Brains were sectioned into the prefrontal cortex (PFC), midbrain, and the hindbrain containing the locus coeruleus (pons/LC). Expression of GAPDH, as a representative gene for numbers of astrocytes, oligodendrocytes, microglia, and neurons shown relative to the neuronal marker MAP2. dCq is difference in Cq quantification cycle between GAPDH and MAP2, ±SEM shown, n=5, Student’s t test comparing TINEVs and control EVs for the regions: p = 0.86, 0.28, and 0.26, respectively.


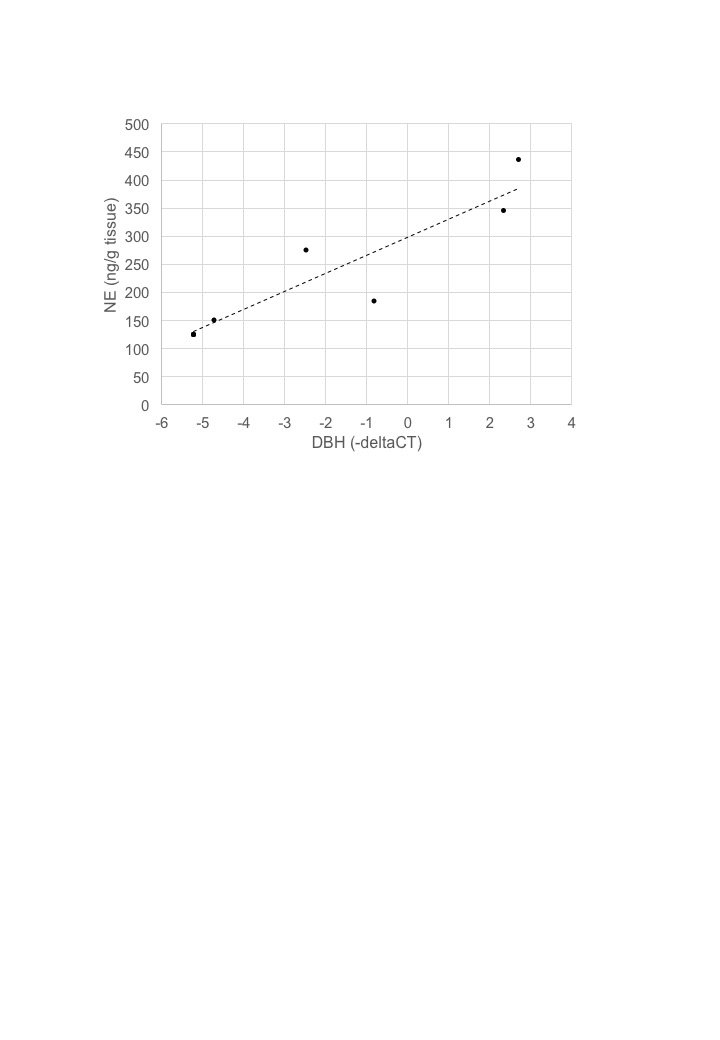


**Figure S4: Dopamine β-hydroxylase during infection directly correlates with norepinephrine (NE) levels in the brain.** Norepinephrine levels were measured by HPLC-ED and dopamine β-hydroxylase (DBH) was quantitated by RT-qPCR (relative to GAPDH) in brain tissue from rats. The Pearson's correlation coefficient is 0.81 and the R value is 0.90 (p=0.014).

**
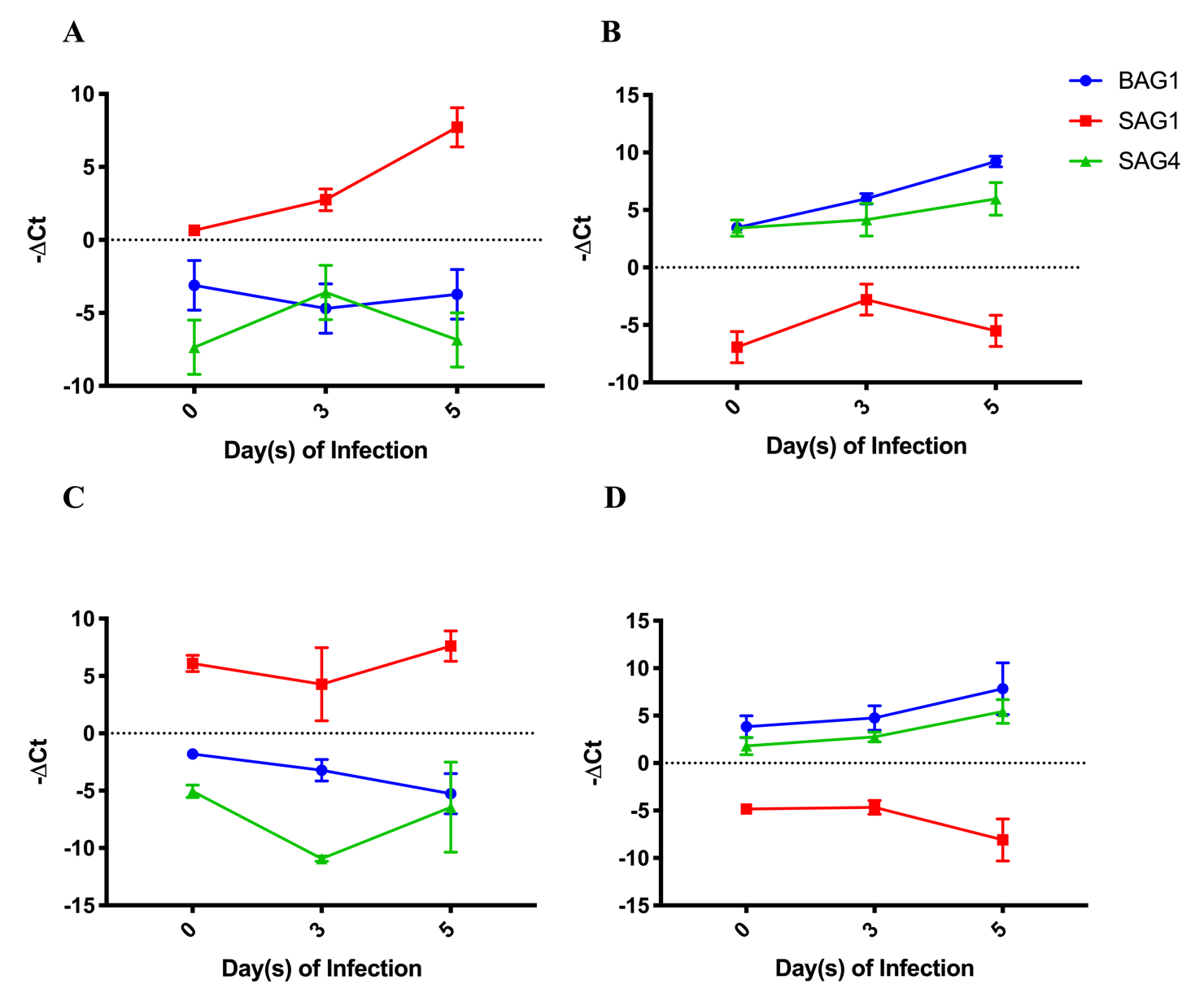
**

**Figure S5: pH shocking of liberated *T. gondii* induced a bradyzoite-like phenotype.** All plots show expression of the bradyzoite markers BAG1 (blue) and SAG4 (green), as well as the tachyzoite marker SAG1 (red) expressed in relation to the housekeeping gene GAPDH over a time course of 5 days of infection. **A)** Standard infection with tachyzoites with RNA collected over a time course of 5 days post-infection of rat catecholaminergic PC12 cells. ±SEM shown, n=6. **B)** Infection of Infection with pH-shocked *T. gondii*, as described in the Methods, of PC12 cells and RNA harvesting for RT-qPCR over a 5 days time course. ±SEM shown, n=6. **C)** Standard infection with tachyzoites as in A of human neuronal M17 cells and sample collection and processing. ±SEM shown, n=6. **D)** Human neuronal M17 cells were infected with pH shocked *T. gondii* and cultured for 5 days. RNA was collected on day(s) 0, 3 and 5; RT-qPCR was then performed ±SEM shown, , n=6.

TSS

UTR

DBH coding seq

**Figure S6: Methylation in the *DBH* upstream region based on bisulfite sequencing.** Graph depicting the changes in CpG methylation across the *DBH* gene region from massively parallel genome sequencing for infected noradrenergic PC12 cells from three infected and three uninfected cultures at day five of *T. gondii* infection aligned with a diagram of the gene.

**Figure S7: UV irradiation reverses the effect of TINEVs on 5’ promoter methylation of *DBH*. A)** A schematic representation of experimental design. TINEVs and EVs derived from PC12 cells were mock or UV irradiated for 10 minutes at 4°C. These were used to treat cells for three days followed by DNA harvesting and methylation analysis. **B)** Methylation of the 5’ region of *DBH*, measured by MSRE qPCR in treated cells. Samples were from untreated cells untreated (grey), cells treated with EVs from uninfected cells (dark blue), TINEVs (labelled iEV; red), UV- irradiated EVs from uninfected cells (light blue), and UV-irradiated TINEVs (dark red); n=6 ±SEM shown. Two-way ANOVA performed, ** p<0.01, *** p<0.001. Tukey’s post hoc test for TINEVs compared to controls and UV treatments , p=0.001; and between controls and UV treatments, p= 0.90.


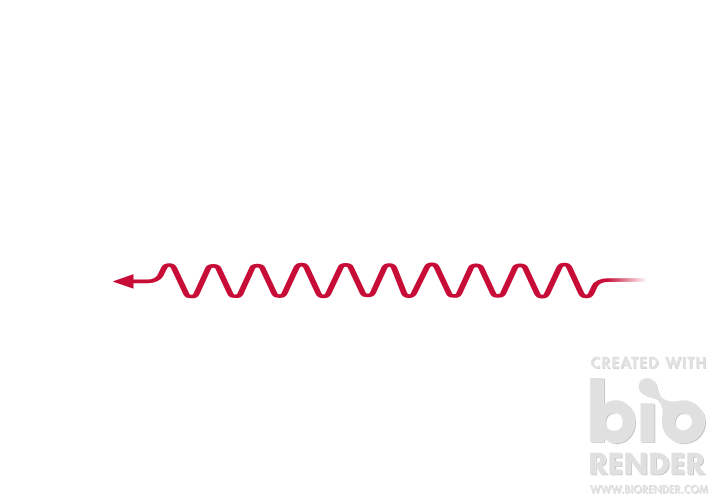


5’

Predicted antisense transcript

TSS

UTR

RT primer 2

-180 -46 +100 +232

-378

Primer set 2

RT primer 3

RT primer 1

Primer set 1

Primer set 4

Primer set 3

A

B


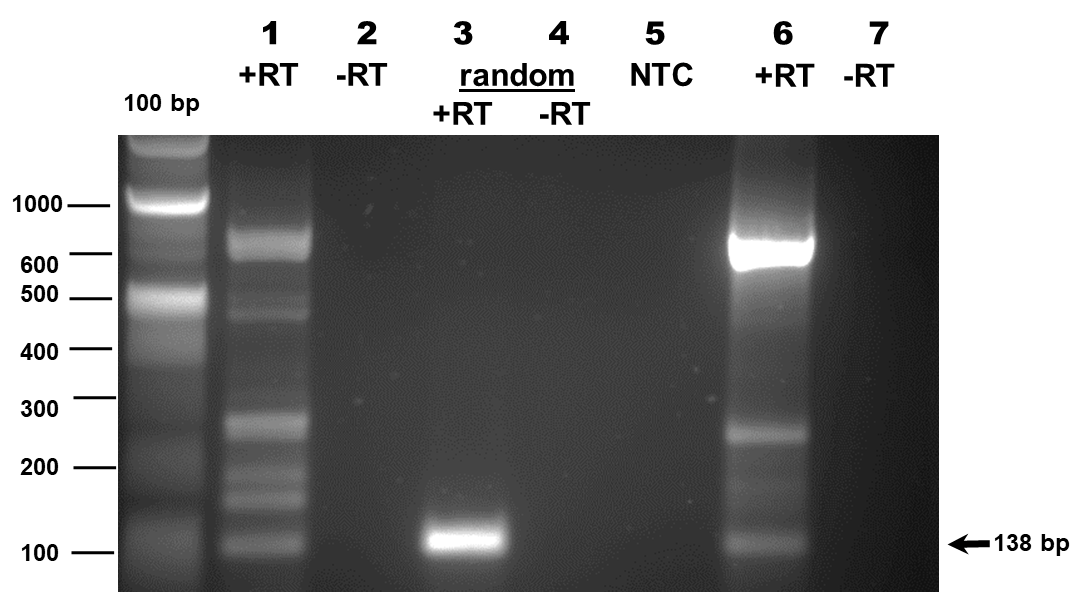

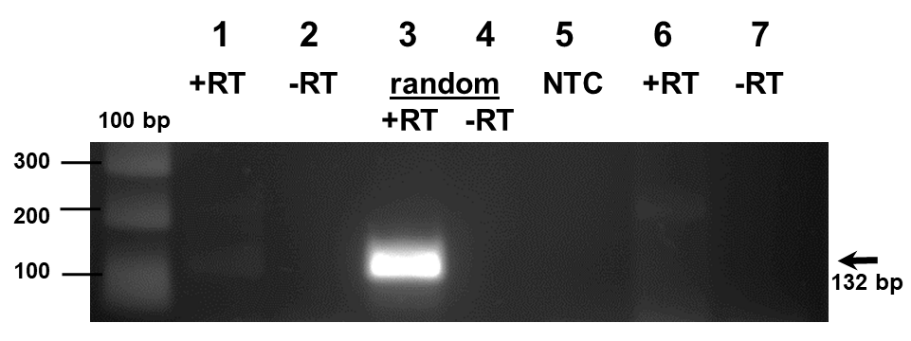


C

D

E


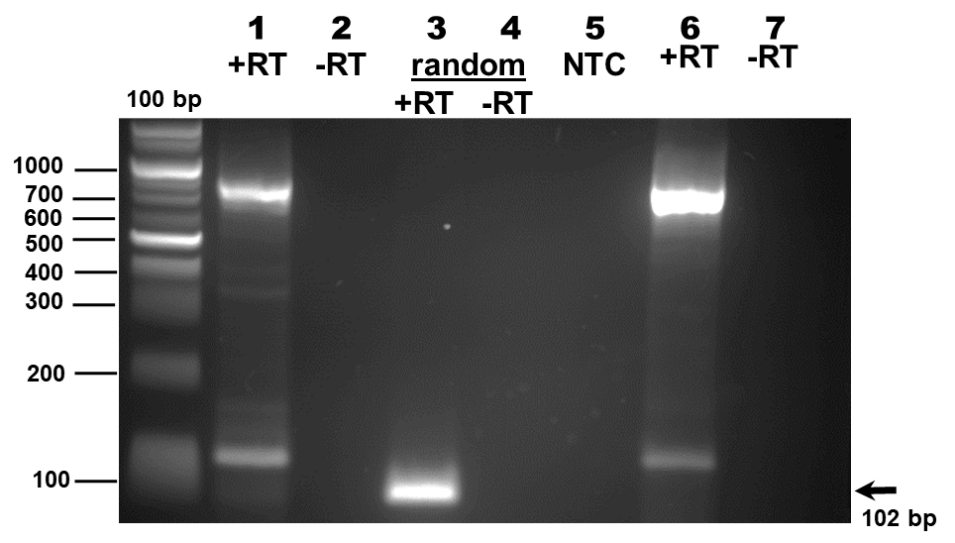

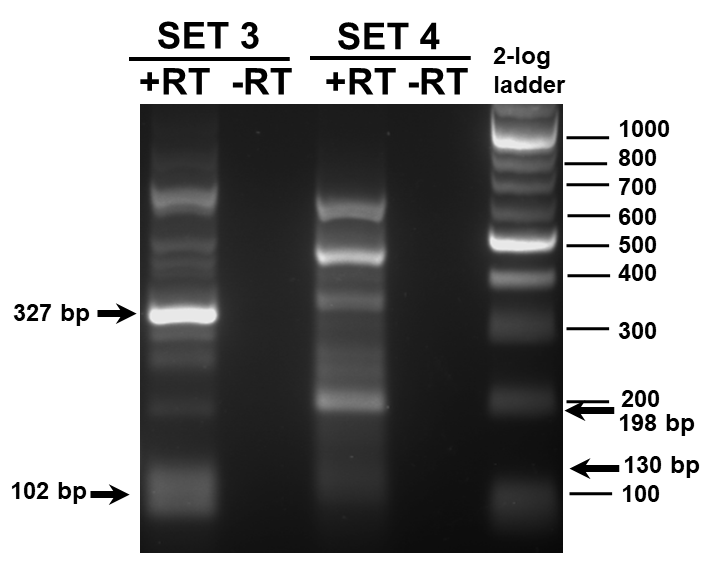


RT primer 1 with PCR Primer set 1

RT primer 2 with PCR Primer set 2

RT primer 3 with PCR Primer set 3 and PCR Primer set 4

RT primer 2 with PCR Primer set 3

**Figure S8: Genome scanning for *DBH* antisense lncRNA**

**A)** Schematic representation of the stepwise walking upstream of the *DBH* gene to identify a putative antisense lncRNA. The binding sites for the directional RT primers for antisense transcript detection are depicted. Primer sets 1-4 were used to amplify cDNAs produced by the directional primers as detailed below. The transcription start site (TSS), untranslated region (UTR) and protein coding region (grey) are highlighted. **B-E)** *DBH* antisense RNA detected in *T. gondii*-infected PC12 cells. Agarose gel resolution of PCR products from directional reverse transcriptase (RT) primers. Control reactions lacking reverse transcriptase (-RT) were performed for each sample. Random hexamer primers that will detect both + and – strands in reverse transcription reactions were also tested. **B)** antisense *DBH* was not detected as a PCR product (132 bp) using directional RT primer 1 (+13) and PCR primer set 1 (+100 to +232) although the random hexamer primers yielded a band indicating *DBH* mRNA. **C)** antisense *DBH* from directional RT primer 2 (-180) was detected by PCR primer set 2 (-46 to +92) as a PCR product (138 bp). Additional bands are visible. **D)** Directional RT primer 3 (-378) detected antisense *DBH* with amplification by PCR primer set 3 (-153 to -51) with a PCR product (102 bp) and by PCR primer set 4 (-310 to -180) with 130 bp PCR product. Unexpected bands at 327 bp and 198 bp are due to amplification using the RT primer 3 and reverse primers for PCR primer set 3 and 4, respectively. **E)** First strand synthesis with RT primer 2 and PCR with primer set 3 yielded an unexpected band at 129 bp rather than the predicted 102 bp band. This was likely due to PCR amplification utilizing RT primer 2 and the reverse primer in set 3. PCR products of bands on gels were confirmed by DNA sequencing.

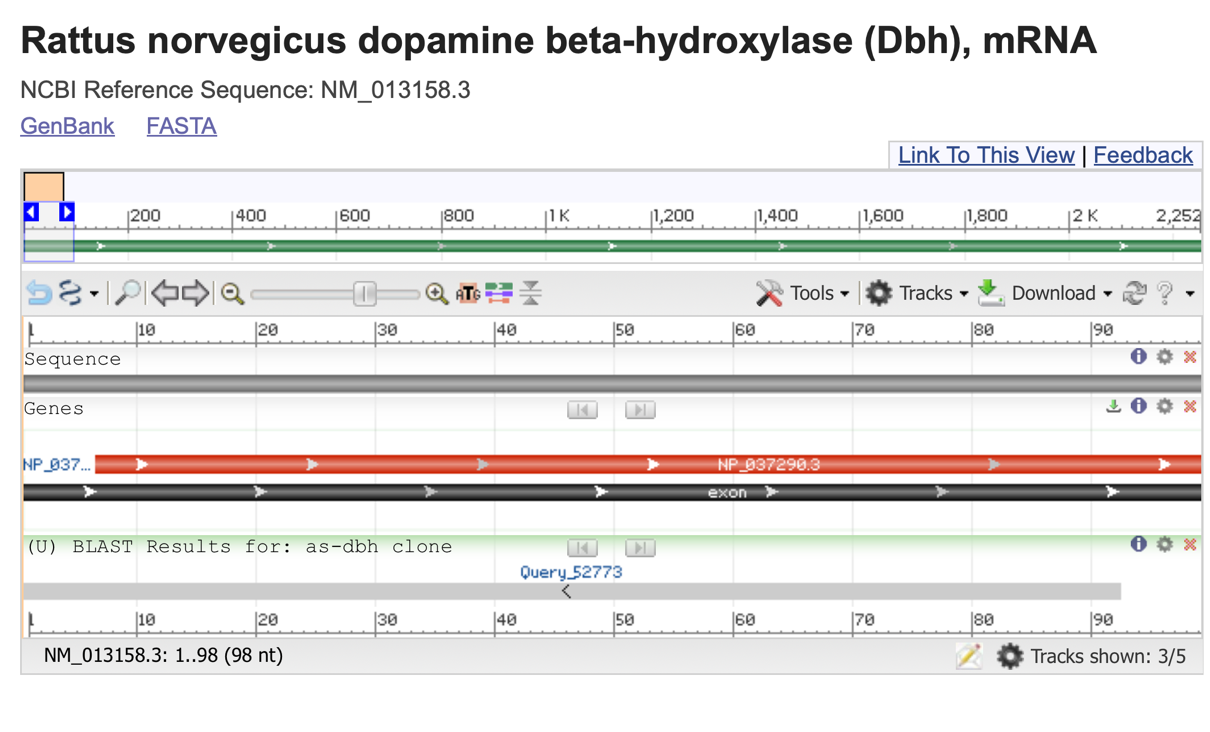


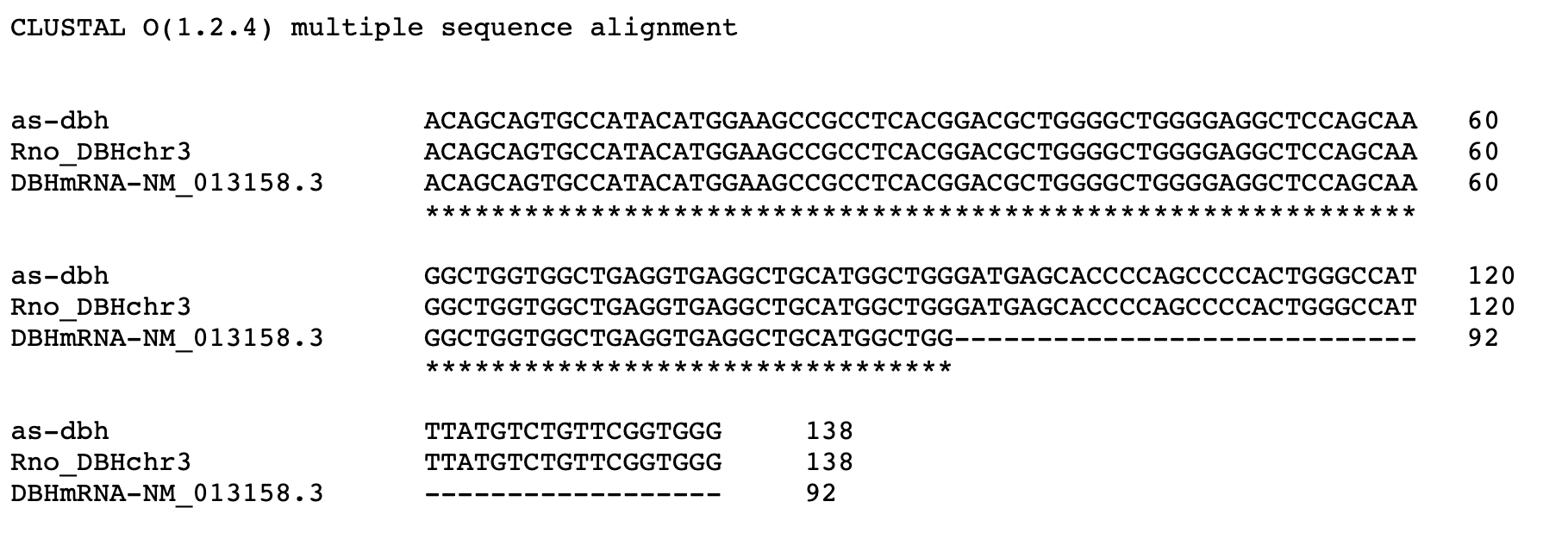


**Figure S9: Controls for qRT-PCR of antisense DBH lncRNA.** Reverse transcription was performed with RNA purified from EVs from *T. gondii*-infected and uninfected PC12 cells with subsequent PCR of cDNA. The predicted size of 138 bp was observed in the iEV (TINEV) sample. This was confirmed by sequencing as shown below. A spurious smaller band occurred randomly in controls without reverse transcriptase (-RT) and with controls that used random hexamer primers for cDNA synthesis as background. Sequencing the antisense DBH lncRNA. The higher band on the agarose gel was extracted and cloned by TopoTA cloning for sequencing. Clones contained a 138 bp insert as predicted. The sequence aligned 100% with the *Rattus norvegicus* genomic sequence and the non-coding, reverse complement of the 5’ end of DBH mRNA (NM_013158.3). Note that the ORF is nucleotides 86 to 1.


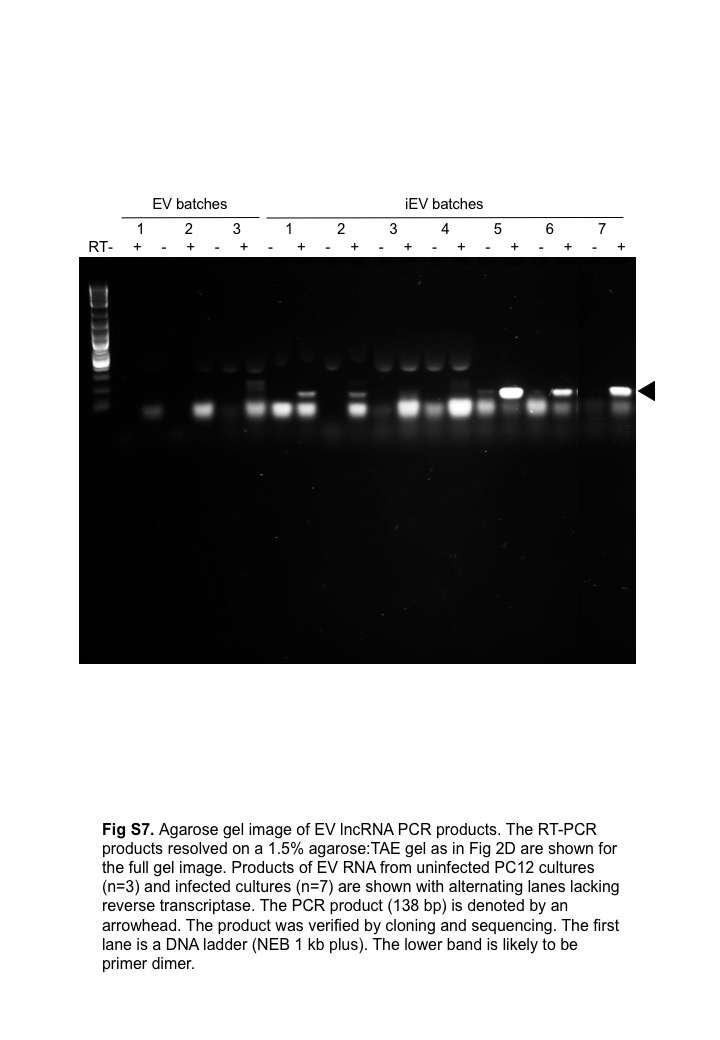


**Figure S10: Agarose gel image of antisense *DBH* detection in TINEVs.** The RT-PCR products resolved on a 1.5% agarose:TAE gel as in Fig 6D are shown with the full gel image. Antisense *DBH* from EVs from uninfected PC12 cultures (n=3) and TINEVs preparations (n=7) are shown with alternating lanes lacking reverse transcriptase. The PCR product (138 bp) is denoted by an arrowhead. The product was verified by cloning and sequencing. The first lane is a DNA ladder (NEB 1 kb plus). A lower (≈100 bp) background band appeared in some lanes.

**SUPPLEMENTAL DATA TABLE**

**1: Table of proteins identified by proteomic analysis. The chart shows the representation of proteins differentially found in the TINEVs. *T. gondii* proteins identified are shown in the table.**

| microneme protein MIC2 |
| --- |
| beta-tubulin |
| elongation factor 1-alpha |
| heat shock protein HSP70 |
| G-protein beta WD-40 repeat containing protein |
| cell division protein CDC48CY |

**2: Primer sequences used throughout.**

| Name | | Sequence |
| --- | --- | --- |
| Rodent DBH | F | 5’-CCACAATCCGGAATATA-3’ |
|  | R | 5’-GATGCCTGCCTCATTG-3’ |
| Human DBH | F | 5’-GAAACGACTCCTCAGGCAT-3’ |
|  | R | 5’-TCGCAGAGTAGAGTGCACA-3’ |
| Rodent MAP2 | F | F 5’-AAGGTGGTGGACGTGTG-3’ |
|  | R | 5’-CTGGTTCATTGCCATTCTC-3’ |
| Rodent GAPDH | F | 5’-GTGGACCTCATGGCCTACAT-3’ |
|  | R | 5’-TGTGAGGGAGATGCTCAGTG-3’ |
| Human GAPDH | F | 5’-AGGGCTGCTTTTAACTCTG-3’ |
|  | R | 5’-CCCCACTTGATTTTGGAG-3’ |
| Rat DBH MSRE | F | 5’-CAGGACGTAGCCACAGATGG-3’ |
|  | R | 5’-TGCCTTCCCCGGG-3’ |
| Human DBH MSRE | F | 5’-CAATACCCCAGAGGGGAGAG-3’ |
|  | R | 5’-GCTTTCCCATCAGGACATGC-3’ |
| Primer Set 1 | F | 5’-CCCACCGAACAGACATAA-3’ |
|  | R | 5’-ACAGCAGTGCCATACAT-3’ |
| Primer Set 2 | F | 5’-TCCTGGTCATCCTGGTG-3’ |
|  | R | 5’-GGATGATCTCCTGGTCATAG-3’ |
| Primer Set 3 | F | 5’-GAGGATCGGAGCAAAGT-3’ |
|  | R | 5’-GCCAGGCTGTACCTAAT-3’ |
| Primer Set 4 | F | 5’-TATGAGGCATTCAGCAG-3’ |
|  | R | 5’-CATCATCAAGAGAATCGAAG-3’ |
| RT-Primer 1 (lncRNA DBH) | F | 5’-CCTCACCTCAGCCACCAG-3’ |
| RT-Primer 2 (lncRNA DBH) | F | 5’-TCCATGCGTCATTAGTGTCAA-3’ |
| RT-Primer 3 (lncRNA DBH) | F | 5’-CACTCACTGTCACTCAGGAAGG-3’ |
